# Population-based rates, timing, and causes of maternal deaths, stillbirths, and neonatal deaths in south Asia and sub-Saharan Africa: a multi-country prospective cohort study

**DOI:** 10.1016/S2214-109X(18)30385-1

**Published:** 2018-10-22

**Authors:** The Alliance for Maternal and Newborn Health Improvement (AMANHI) mortality study group, Imran Ahmed, Said Mohammed Ali, Seeba Amenga-Etego, Shabina Ariff, Rajiv Bahl, Abdullah H Baqui, Nazma Begum, Nita Bhandari, Kiran Bhatia, Zulfiqar A Bhutta, Godfrey Biemba, Saikat Deb, Usha Dhingra, Brinda Dube, Arup Dutta, Karen Edmond, Fabian Esamai, Wafaie Fawzi, Amit Kumar Ghosh, Peter Gisore, Caroline Grogan, Davidson H Hamer, Julie Herlihy, Lisa Hurt, Muhammad Ilyas, Fyezah Jehan, Michel Kalonji, Jasmine Kaur, Rasheda Khanam, Betty Kirkwood, Aarti Kumar, Alok Kumar, Vishwajeet Kumar, Alexander Manu, Irene Marete, Honorati Masanja, Sarmila Mazumder, Usma Mehmood, Shambhavi Mishra, Dipak K Mitra, Erick Mlay, Sanjana Brahmawar Mohan, Mamun Ibne Moin, Karim Muhammad, Alfa Muhihi, Samuel Newton, Serge Ngaima, Andre Nguwo, Imran Nisar, Maureen O'Leary, John Otomba, Pawankumar Patil, Mohammad Abdul Quaiyum, Mohammed Hefzur Rahman, Sunil Sazawal, Katherine EA Semrau, Caitlin Shannon, Emily R Smith, Sajid Soofi, Seyi Soremekun, Venantius Sunday, Sunita Taneja, Antoinette Tshefu, Yaqub Wasan, Kojo Yeboah-Antwi, Sachiyo Yoshida, Anita Zaidi

## Abstract

**Background:**

Modelled mortality estimates have been useful for health programmes in low-income and middle-income countries. However, these estimates are often based on sparse and low-quality data. We aimed to generate high quality data about the burden, timing, and causes of maternal deaths, stillbirths, and neonatal deaths in south Asia and sub-Saharan Africa.

**Methods:**

In this prospective cohort study done in 11 community-based research sites in south Asia and sub-Saharan Africa, between July, 2012, and February, 2016, we conducted population-based surveillance of women of reproductive age (15–49 years) to identify pregnancies, which were followed up to birth and 42 days post partum. We used standard operating procedures, data collection instruments, training, and standardisation to harmonise study implementation across sites. Verbal autopsies were done for deaths of all women of reproductive age, neonatal deaths, and stillbirths. Physicians used standardised methods for cause of death assignment. Site-specific rates and proportions were pooled at the regional level using a meta-analysis approach.

**Findings:**

We identified 278 186 pregnancies and 263 563 births across the study sites, with outcomes ascertained for 269 630 (96·9%) pregnancies, including 8761 (3·2%) that ended in miscarriage or abortion. Maternal mortality ratios in sub-Saharan Africa (351 per 100 000 livebirths, 95% CI 168–732) were similar to those in south Asia (336 per 100 000 livebirths, 247–458), with far greater variability within sites in sub-Saharan Africa. Stillbirth and neonatal mortality rates were approximately two times higher in sites in south Asia than in sub-Saharan Africa (stillbirths: 35·1 per 1000 births, 95% CI 28·5–43·1 *vs* 17·1 per 1000 births, 12·5–25·8; neonatal mortality: 43·0 per 1000 livebirths, 39·0–47·3 *vs* 20·1 per 1000 livebirths, 14·6–27·6). 40–45% of pregnancy-related deaths, stillbirths, and neonatal deaths occurred during labour, delivery, and the 24 h postpartum period in both regions. Obstetric haemorrhage, non-obstetric complications, hypertensive disorders of pregnancy, and pregnancy-related infections accounted for more than three-quarters of maternal deaths and stillbirths. The most common causes of neonatal deaths were perinatal asphyxia (40%, 95% CI 39–42, in south Asia; 34%, 32–36, in sub-Saharan Africa) and severe neonatal infections (35%, 34–36, in south Asia; 37%, 34–39 in sub-Saharan Africa), followed by complications of preterm birth (19%, 18–20, in south Asia; 24%, 22–26 in sub-Saharan Africa).

**Interpretation:**

These results will contribute to improved global estimates of rates, timing, and causes of maternal and newborn deaths and stillbirths. Our findings imply that programmes in sub-Saharan Africa and south Asia need to further intensify their efforts to reduce mortality rates, which continue to be high. The focus on improving the quality of maternal intrapartum care and immediate newborn care must be further enhanced. Efforts to address perinatal asphyxia and newborn infections, as well as preterm birth, are critical to achieving survival goals in the Sustainable Development Goals era.

**Funding:**

Bill & Melinda Gates Foundation.

## Introduction

Global estimates of the burden, causes, and timing of maternal deaths, stillbirths, and neonatal deaths have been instrumental in setting scientific, programmatic, and policy agendas, and in tracking progress towards the Millennium Development Goals (MDGs) and Sustainable Development Goals (SDGs). In the absence of complete vital registration data from low-income and middle-income countries, the burden of estimates of maternal, newborn, and stillbirth mortality rely on models that use data from diverse sources such as population-based surveys, civil registration, and country censuses.^1^, ^2^, ^3^, ^4^, ^5^, ^6^

Despite major efforts to pool available data, modelled estimates to date are likely to be inaccurate. The data sources used are known to be of variable quality because of methodological limitations, including study design (eg, cross-sectional surveys), long recall periods, misclassification of timing of key outcomes, lack of standardised case definitions, and inclusion of studies that do not provide population-based mortality data. These data gaps are greatest in south Asia and sub-Saharan Africa. Despite reported declines, maternal and neonatal mortality rates remain unacceptably high in these regions^7^ and most countries in south Asia and sub-Saharan Africa failed to meet the MDGs on maternal and child survival.^8^, ^9^, ^10^ About three-quarters of the estimated global stillbirths occur in south Asia and sub-Saharan Africa; however, these estimates are based on sparse data.^3^

**Research in context**

**Evidence before this study**

Global and regional maternal, neonatal, and stillbirth mortality estimates rely on models that use data from diverse sources, mostly surveys. The data sources are known to be of variable quality and have methodological limitations. We searched PubMed, websites of UN agencies (WHO, UNICEF, and the UN Population Fund) using the following strategy: (global OR regional OR world) AND (maternal OR neonatal OR newborn OR stillbirth) AND (mortality OR death) AND (estimate OR level OR trend OR cause OR burden). We identified estimates issued by UN inter-agency groups, the Institute for Health Metrics and Evaluation, the Maternal and Child Epidemiology Estimation Group (previously the Child Health Epidemiology Reference Group), and the London School of Hygiene & Tropical Medicine. High maternal mortality ratios, stillbirth rates, and neonatal mortality rates were reported in both south Asia and sub-Saharan Africa, with higher rates in sub-Saharan Africa. The three most common causes of maternal death were shown to be indirect medical causes, haemorrhage, and hypertensive disorders of pregnancy; causes of stillbirth at population level are unavailable. Preterm birth is known to be the most common cause of neonatal deaths, and deaths from infection and asphyxia are decreasing.

**Added value of this study**

This study provides high-quality primary data about the population-based burden, timing, and causes of maternal deaths, stillbirths, and neonatal deaths from multiple sites in sub-Saharan Africa and south Asia. Maternal mortality ratios, stillbirth rates, and neonatal mortality rates are higher in south Asia than the published global and regional estimates. The proportion of maternal and neonatal deaths that occur around the time of birth are even higher than published estimates. However, antepartum stillbirths are more common than are intrapartum stillbirths, even in high mortality regions in south Asia and sub-Saharan Africa. Our study corroborates the cause of death distribution of maternal death from published estimates. To our knowledge, this is the largest dataset about causes of stillbirth, approximately 90% of which were related to maternal health. The most common causes of antepartum stillbirths include hypertensive disorders of pregnancy, infections, and placental complications resulting in antepartum haemorrhage. Complications of labour and delivery, hypertensive disorders, and antepartum haemorrhage accounted for most intrapartum stillbirths.

**Implications of all the available evidence**

Inclusion of our data will substantially improve the quality of input data for modelling required to develop global estimates of maternal death, neonatal deaths, and stillbirths. Our findings have several implications for public health programmes in both south Asia and sub-Saharan Africa. These implications include increased focus on reduction in stillbirth through improved maternal care during the last trimester of pregnancy, labour, and birth. The latter, along with neonatal care, is crucial to reducing the large number of maternal and early neonatal deaths. Finally, our findings indicate that the highest attention needs to be given to interventions that reduce perinatal asphyxia and neonatal infection related deaths, in addition to preterm birth complications.

Thus, there is an urgent need for high-quality, standardised, population-based, prospective data to quantify the burden, causes, and timing of maternal and newborn deaths and stillbirths. The Alliance for Maternal and Newborn Health Improvement (AMANHI) designed a multi-country population-based cohort study to address this data gap. The study involved active identification and prospective follow-up of pregnant women through the postpartum period in 11 community-based research sites in south Asia and sub-Saharan Africa. Our aim was to empirically determine the population-based burden, timing, and causes of maternal deaths, stillbirths, and neonatal deaths in these sites.

## Methods

### Study design and participants

Detailed methods of this study have been published previously.^11^ The AMANHI mortality study started in July, 2012, and ended in February, 2016, and was done in five sites in south Asia (Bangladesh [Sylhet], India [Haryana, Uttar Pradesh], and Pakistan [Karachi, Matiari]) and six sites in sub-Saharan Africa (Democratic Republic of Congo [North and South Ubangi], Ghana [Brong Ahafo], Kenya [Western Province], Tanzania [Ifakara, Pemba], and Zambia [Southern Province]). Population-based surveillance of approximately 2 million women of reproductive age was done every 2–3 months to identify pregnancies. The cohort of pregnant women who gave consent was followed up from birth to 42 days post partum.

The study was done in predominantly rural communities, except for Karachi, Pakistan, which was periurban-urban. When the data collection for this study started, all sites had ongoing studies of neonatal health in various stages of implementation. A description of individual sites is provided in the appendix. A uniform protocol for this study was implemented across sites.^11^ Two joint training workshops were held to harmonise the implementation of study procedures, including data collection and physician coding for verbal autopsies.

All sites except Zambia already had population-based surveillance of all women of reproductive age in geographically defined areas. Trained field workers visited all women of reproductive age in their study areas at home every 2–3 months to collect baseline sociodemographic data, and information about all adult female deaths, pregnancies, and pregnancy outcomes, including deaths from conception to 42 days post partum. In Zambia, pregnancies were identified at the time of first booking at antenatal care clinics and then followed up at home.

We used standardised procedures and questionnaires based on a set of common core variables across all sites. The data management system at each site had range, logical, and consistency checks built into the software. The project team reviewed the data on a regular basis and transferred the data quarterly to a coordinating team at WHO Geneva for further checks. The WHO team did six monthly site visits to monitor quality of field implementation and data.

The study received ethics clearance from institutional review boards and ethics review committees in the participating countries, host institutions of principal investigators, and WHO. All participants provided informed written consent before inclusion in the study.

### Procedures

Verbal autopsy is a method for ascertaining causes of death on the basis of an interview with caregivers regarding their knowledge of the symptoms, signs, and circumstances preceding death. Verbal autopsy was done for deaths of all women of reproductive age, neonatal deaths, and stillbirths identified during surveillance at each site.^12^, ^13^, ^14^ We created a table of common core verbal autopsy variables, based on WHO verbal autopsy and InterVA standard tools,^15^, ^16^ and translated this into site-specific verbal autopsy questionnaires. The verbal autopsy in all sites included three sections: a narrative of the circumstances leading to death and timing of death, closed-ended questions, and review of written health records. The purpose of using the verbal autopsy tool adapted for this study was to ascertain if an adult female death was pregnancy-related or not, to discriminate a stillbirth from an early neonatal death, and to ascertain causes of stillbirths, maternal, and newborn deaths.

The verbal autopsy respondent was an adult who lived closely with the deceased in the period immediately preceding the death and could provide information about the circumstances leading to death. For stillbirths and neonatal deaths, the preferred respondent was the mother, except in circumstances when she was not alive at the time of the interview. For maternal deaths, respondents were the mothers-in-law or husbands. Master trainers from all sites were trained in the core AMANHI surveillance and verbal autopsy training procedures during two training workshops at WHO Geneva. The master trainers then trained verbal autopsy data collectors. A random selection of 5% of the verbal autopsy interviews was directly observed and feedback was provided to the data collectors on their performance. Physician verbal autopsy coders had thorough training, during which principles of the International Classification of Diseases (ICD) were used; they went through an accreditation process overseen by WHO.

We developed a coding manual and a specific coding software for the AMANHI study, based on the hierarchical ICD-10 classification system^17^, ^18^ and the WHO Verbal Autopsy Coding Standards,^19^ including a list of programmatically relevant causes of maternal, fetal, and neonatal death. In this system, cause of death was considered to be the condition that occurred earliest and started the chain of events that resulted in death. We developed a web-based data management tool that included all of the information from the verbal autopsy forms and used the hierarchical framework for coding. At least two trained, accredited physicians independently coded all verbal autopsies, with a third or fourth physician reviewing the verbal autopsy when previous coders did not agree. A final cause of death was assigned when at least two of the physicians agreed. The software calculated the frequency of agreement between physicians and the final cause of death code.

### Statistical analysis

The sample size was determined before the study. We estimated that a total of 263 000 pregnant women would provide estimates of the rarest outcome (maternal mortality) with a relative precision of 8% for sub-Saharan Africa and 10% for south Asia, based on a 95% CI. This sample size would also allow us to quantify single causes that accounted for at least 20% of maternal mortality with 15% relative precision, as well as giving a higher degree of precision for regional and country estimates of more common outcomes such as stillbirths and neonatal deaths.

We did all analyses using Stata (version 14). To calculate maternal mortality ratio, neonatal mortality rate, and stillbirth rate by region, we pooled site-specific estimates using a random effects meta-analysis with DerSimonian and Laird inverse variance weights.^20^

We did simple tabulations to describe the timing and causes of pregnancy-related deaths, stillbirths, and neonatal deaths for each site. We estimated the overall and regional time-specific and cause-specific mortality fractions for maternal, fetal, and neonatal deaths using one-stage meta-analyses with multinomial logit models and random effects for site.

We did ecological analyses to explore whether any differences in stillbirths or neonatal mortality rates between sites could be explained by differences in the proportion of births occurring in health facilities, because a large proportion of these deaths occur around the time of delivery. We did similar analyses using proportion of mothers with schooling as a general indicator of development. We did not do these analyses for maternal mortality ratio because of the wide CIs for estimates of maternal mortality.

Event definitions and summary measure definitions were standard, as defined by WHO (appendix). We enumerated all deaths among women of reproductive age. We defined maternal death as a pregnancy-related death from any cause related to or aggravated by the pregnancy or its management, but not from accidental or incidental causes. For calculation of maternal mortality ratio, we obtained the number of maternal deaths by multiplying the number of all pregnancy-related deaths in a site by the proportion of non-accidental deaths among those with completed verbal autopsies in that site. We assumed that pregnancy-related deaths for which the cause of death was indeterminate were not accidental deaths, because this cause of death is relatively easy to determine. We did not estimate the maternal mortality ratio for the Zambia site because surveillance only covered pregnant women who had already reported to an antenatal clinic; any deaths early in pregnancy would therefore not have been captured. By contrast, all other sites conducted a verbal autopsy for all deaths of all women of reproductive age.

Timing of pregnancy-related deaths was determined from the verbal autopsy with date of last menstrual period (when available) or reported duration of pregnancy at the time of death, date of delivery (in case death occurred after delivery), and date of death. Stillbirth timing was ascertained by physicians who reviewed the verbal autopsy, on the basis of the open narrative and specific questions about fetal movements during labour, fetal heart-rate recording, and whether the stillbirth was fresh or macerated. Physicians ascertained the difference between a miscarriage and a stillbirth by gestational age at the time when pregnancy ended. If gestational age when pregnancy ended was less than 28 weeks, it was coded as an abortion. If it was 28 weeks or longer, it was coded as a stillbirth. We calculated timing of neonatal deaths from date and time of birth and date and time of death. When neither the time of birth or death was available, the difference between the dates of death and birth was considered to be the age of death. If this difference was 1 day, we used the verbal autopsy narrative to ascertain if the death occurred in the first or the second 24 h of life.

A single underlying cause of death was reported for every death. Directions provided to the physicians for ascertainment of causes of maternal and neonatal deaths and stillbirths are shown in the appendix.

### Role of the funding source

The funder of the study had no role in study design, data collection, data analysis, data interpretation, or writing of the report. The corresponding author had full access to all the data in the study and had final responsibility for the decision to submit for publication.

## Results

**Figure 1:**
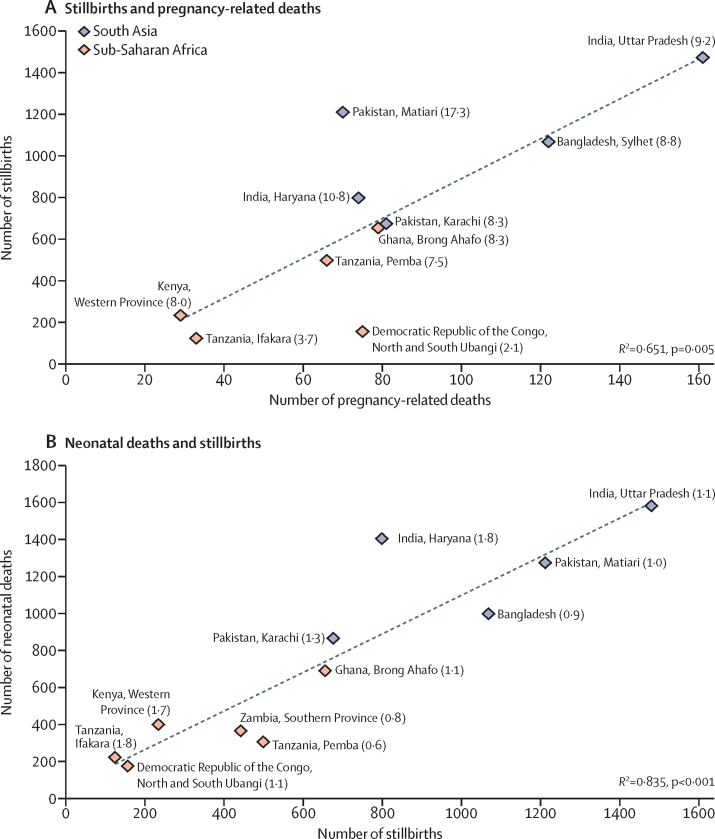
Associations between stillbirths and pregnancy-related deaths (A) and between stillbirths and neonatal deaths (B) Ratios are indicated in parentheses for each site: stillbirths to pregnancy-related deaths (A), and neonatal deaths to stillbirths (B).

We identified 278 186 pregnancies across the study sites, with outcomes ascertained for 269 630 (96·9%), including 8761 (3·2%) that ended in miscarriage or induced abortion (table 1). The remaining 260 869 pregnancies resulted in a total of 263 563 births, with 256 228 liveborn babies and 7335 stillbirths, of which verbal autopsy could be done for 6549, time of death could be ascertained in 6478, and a cause of death could be determined in 4858. There were 812 pregnancy-related deaths (deaths during pregnancy, childbirth, and within 42 days post partum), of which verbal autopsy could be done for 790, time of death could be ascertained in 725, and a cause of death could be determined in 620; 8244 neonatal deaths that occurred from birth up to 28 days after birth. Table 1 also shows the maternal mortality ratio, stillbirth rate, and neonatal mortality rate for each site, together with overall rates in the study sites in south Asia and sub-Saharan Africa; meta-analyses results are shown in figure 1.

**Table 1:** Enrolment, pregnancy outcomes, and rates of maternal and neonatal mortality

		**Pregnancies identified**	**Outcomes ascertained**	**Abortions or miscarriages (pregnancy identification to 28 weeks' gestation)**	**Stillbirths (28 weeks' gestation to birth)**	**Livebirths**	**Pregnancy-related deaths**	**Neonatal deaths**	Maternal mortality ratio (per 100 000 livebirths)*	**Stillbirth rate (per 1000 births)**	**Neonatal mortality rate (per 1000 livebirths)**
Bangladesh		30 253	28 701	1655 (5·8%)	1068	26 295	122	995	456 (375–538)	39·0 (36·7–41·3)	37·8 (35·5–40·2)
India											
	Haryana	38 600	38 240	2765 (7·2%)	798	35 000	74	1399	191 (146–237)	22·3 (20·8–23·8)	40·0 (37·9–42·0)
	Uttar Pradesh	40 738	40 298	1162 (2·9%)	1479	37 813	161	1575	399 (336–463)	37·6 (35·8–39·5)	41·7 (39·6–43·7)
Pakistan											
	Matiari	31 144	28 771	700 (2·4%)	1211	27 062	70	1269	259 (198–319)	42·8 (40·5–45·2)	46·9 (44·4–49·4)
	Karachi	19 790	18 354	663 (3·6%)	675	17 189	81	862	460 (358–561)	37·8 (35·0–40·6)	50·1 (46·9–53·4)
Democratic Republic of the Congo		6407	6330	20 (0·3%)	155	6145	75	173	1188 (917–1459)	24·6 (20·8–28·4)	28·2 (24·0–32·3)
Ghana		25 790	24 857	1063 (4·3%)	654	23 640	79	687	326 (253–398)	26·9 (24·9–29·0)	29·1 (26·9–31·2)
Kenya		31 059	31 023	170 (0·5%)	233	30 992	29	397	94 (60–128)	7·5 (6·5–8·4)	12·8 (11·6–14·1)
Tanzania											
	Ifakara	8881	8253	88 (1·1%)	123	8128	33	221	406 (268–544)	14·9 (12·3–17·5)	27·2 (23·7–30·8)
	Pemba	19 774	19 537	431 (2·2%)	498	18 882	66	303	350 (265–434)	25·7 (23·5–27·9)	16·0 (14·3–17·8)
Zambia		25 750	25 266	44 (0·2%)	441	25 082	22	363	NA†	17·3 (15·7–18·9)	14·5 (13·0–16·0)
South Asia		160 525	154 364	6945 (4·5%)	5231	143 359	508	6100	336 (247–458)‡	35·1 (28·5–43·1)‡	43·0 (39·0–47·3)‡
Sub-Saharan Africa		117 661	115 296	1816 (1·6%)	2104	112 869	304	2144	351 (168–732)‡	17·1 (12·5–25·8)‡	20·1 (14·6–27·6)‡
Overall		278 186	269 630	8761 (3·2%)	7335	256 228	812	8244	..	..	..

Data are n, n (%), rate (95% CI), or ratio (95% CI). NA=not applicable.

* Number of maternal deaths calculated by multiplying the number of all pregnancy-related deaths in a site by the proportion of non-accidental deaths among those with completed verbal autopsies in that site.

† Zambia was excluded from calculation of the maternal mortality ratio because of differences in enrolment strategy of women of reproductive age.

‡ Obtained from meta-analysis.

The pooled maternal mortality ratio for sites in south Asia was 336 per 100 000 livebirths (95% CI 247–458) and in sub-Saharan Africa was 351 per 100 000 livebirths (95% CI 168–732), with overlapping CIs. The maternal mortality ratios in in sub-Saharan African sites varied widely, ranging from 94 per 100 000 livebirths in Kenya to 1188 per 100 000 livebirths in Democratic Republic of the Congo (22 pregnancy-related deaths from Zambia were excluded for reasons previously described; table 1). The variation was smaller in sites in south Asia, ranging from 191 per 100 000 livebirths in Haryana, India to 460 per 100 000 livebirths in Karachi, Pakistan (table 1).

The pooled stillbirth rate for the south Asia sites was about two times higher than that for the sites in sub-Saharan Africa (35·1 per 1000 births, 95% CI 28·5–43·2 *vs* 17·9 per 1000 births, 12·5–25·8), with no overlap in the CIs. The stillbirth rate in the Haryana site (22·3 per 1000 births, 20·8–23·8) was lower than that in other sites in south Asia (table 1). In sub-Saharan Africa, stillbirth rates were lower in Kenya (7·5 per 1000 births, 6·5–8·4), Ifakara, Tanzania (14·9 per 1000 births, 12·5–17·3), and Zambia (17·3 per 1000 births, 15·7–18·9), than in the other three sites (table 1).

The pooled neonatal mortality rate for sites in south Asia was also about two times higher than that for sites in sub-Saharan Africa (43·0 per 1000 livebirths, 95% CI 39·2–47·3 *vs* 20·1 per 1000 livebirths, 14·6–27·6), with no overlap in the CIs. In south Asia, Matiari (46·9 per 1000 livebirths, 44·4–49·4) and Karachi (50·1 per 1000 livebirths, 46·9–53·4), both in Pakistan, had higher neonatal mortality rates than the other three sites (table 1). The variation in sub-Saharan Africa was greater, with much lower neonatal mortality rates in Kenya (12·8 per 1000 livebirths, 11·6–14·1), Zambia (14·5 per 1000 livebirths, 13·0–16·0), and Pemba, Tanzania (16·0 per 1000 livebirths, 14·3–17·8) than the other three sites (table 1).

There was a strong correlation (*R*^2^=0·651, p=0·005) between the numbers of pregnancy-related deaths and stillbirths in each site (figure 1A). The median was 8·3 stillbirths for each pregnancy-related death (IQR 7·5–9·2), but the ratios were all higher in the south Asian sites (ranging from 8·3 to 17·3 stillbirths per pregnancy-related death) than in the sub-Saharan Africa sites, where the range was 2·1 to 8·3 stillbirths per pregnancy-related death. There was also a strong correlation (*R*^2^=0·835, p<0·0001 between the numbers of neonatal deaths and stillbirths in each site (figure 1B), with a median of 1·1 neonatal deaths for each stillbirth (IQR 0·9–1·7). These ratios were similar in sites in south Asia (range 0·9–1·8) and sub-Saharan Africa (range 0·6–1·8).

**Figure 2:**
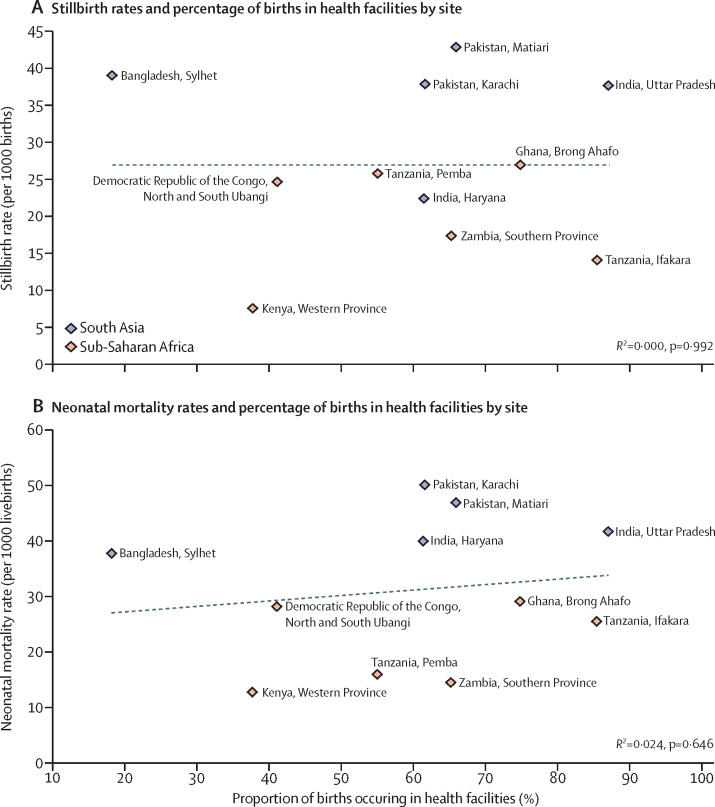
Associations between stillbirth rate and proportion of births in health facilities (A) and between neonatal mortality rate and proportion of births in health facilities (B)

**Figure 3:**
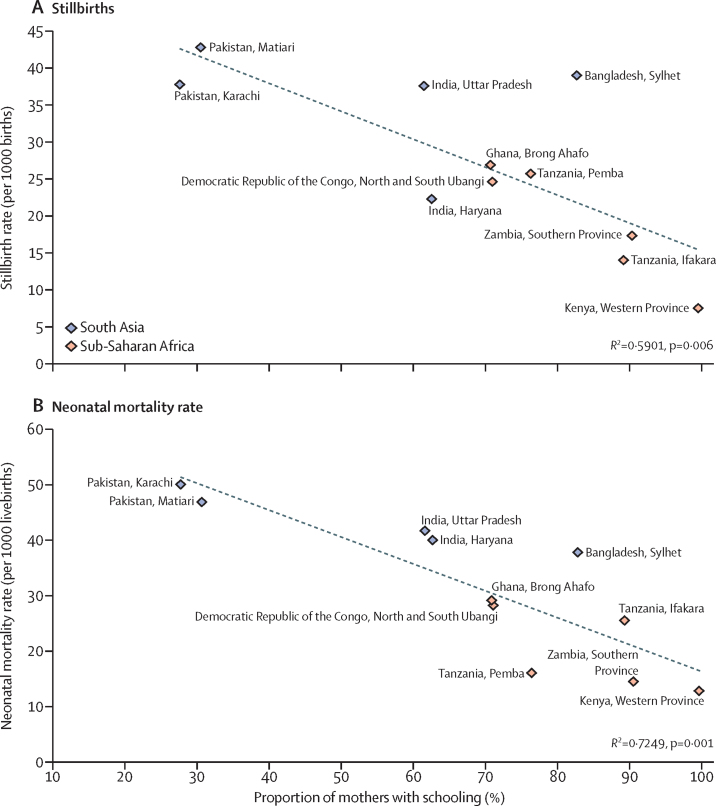
Associations between stillbirth rate and proportion of mothers with any schooling (A) and between neonatal mortality rate and proportion of mothers with any schooling (B)

The population-based proportion of facility births in the study sites ranged from 20% to 87%. When we plotted site-specific stillbirth rates and neonatal mortality rates against population-based proportion of facility births in the same site, there were no correlations (stillbirth rate *R*^2^<0·001, p=0·992; neonatal mortality rate *R*^2^=0·024, p=0·646; figure 2). By contrast, there were strong correlations between the site-specific stillbirth rate (*R*^2^=0·5901; p=0·006) and the neonatal mortality rate (*R*^2^=0·725; p=0·001) when we plotted them against the population-based proportion of mothers with any schooling (figure 3).

**Figure 4:**
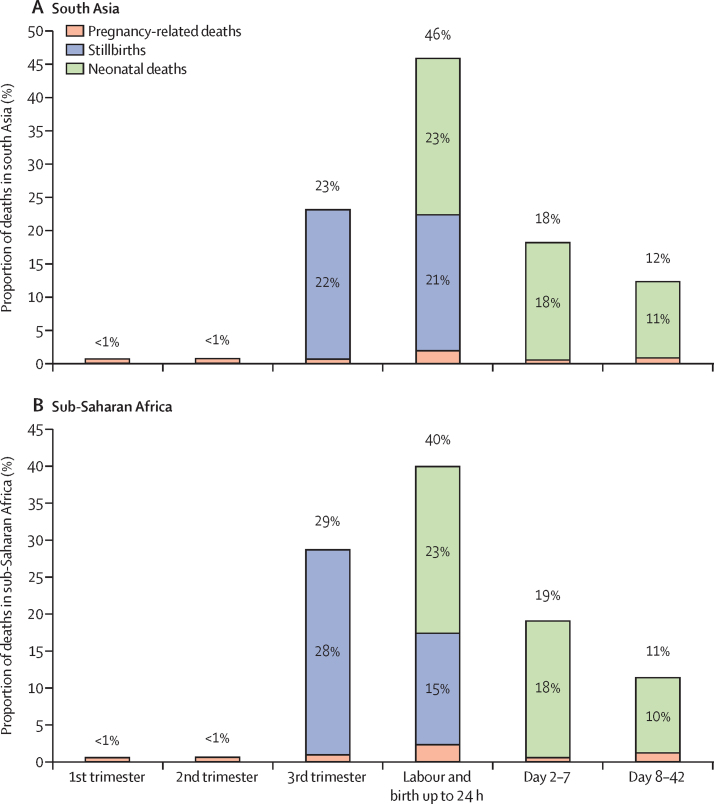
Timing of pregnancy-related deaths, stillbirth, and neonatal deaths in each region

Of the 790 pregnancy-related deaths (excluding Zambia), timing of death was known for 725 (92%) deaths and verbal autopsies were done for 681 (86%) deaths. The biggest burden of deaths occurred during labour, delivery, and the first 24 h in sites in both south Asia (44%, 95% CI 40–49) and sub-Saharan Africa (40%, 34–46). About a fifth of deaths occurred after the first week of birth, between days 8 and 42 (figure 4, appendix).

**Figure 5:**
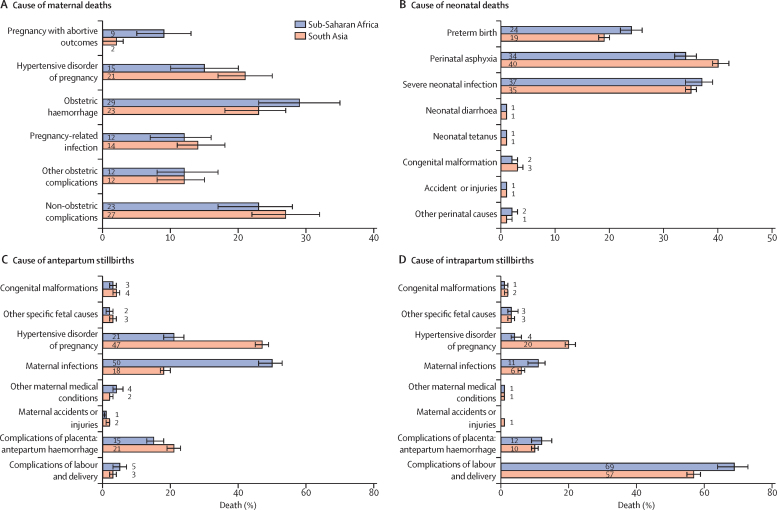
Causes of pregnancy-related death (A), neonatal deaths (B), antepartum stillbirths (C), and intrapartum stillbirths (D) in each region

In all sites, 23 (3%) of the 681 pregnancy-related deaths with verbal autopsies were determined to be due to coincidental causes (accidental or incidental causes); the remaining 658 (97%) were considered to be maternal deaths. A cause of death was assigned for 597 (91%) of these deaths (table 2). Obstetric haemorrhage (south Asian sites 23%, 95% CI 19–28; sub-Saharan African sites 29%, 23–35), non-obstetric complications as the indirect cause of maternal deaths (south Asian sites 27%, 22–32; sub-Saharan African sites 23%, 18–29), and hypertensive disorders of pregnancy, childbirth, and puerperium (south Asian sites 21%, 17–25; sub-Saharan African sites 15%, 10–20) were the three most common causes of maternal deaths (table 2). Pregnancy-related infection and other obstetric complications were the next two most common causes, each accounting for more than 10% of maternal deaths. These five causes of death accounted for more than 90% of all maternal deaths in south Asia and sub-Saharan Africa (table 2; figure 5).

**Table 2:** Causes of pregnancy-related and maternal deaths, by site (excluding Zambia*)

		**Pregnancy-related deaths**	**Verbal autopsy done**	**Coincidental causes**	**Maternal deaths**	**Maternal deaths with cause determined**†						
						Total	Pregnancy with abortive outcomes	Hypertensive disorders in pregnancy, childbirth, and puerperium	Obstetric haemorrhage	Pregnancy-related infection	Other obstetric complications	Indirect cause of maternal deaths: non-obstetric complications
Bangladesh		122	81 (66%)	1 (1%)	80	70	2 (3%)	23 (33%)	16 (23%)	6 (9%)	15 (21%)	8 (11%)
India												
	Haryana	74	68 (92%)	6 (10%)	62	53	1 (2%)	13 (25%)	9 (17%)	14 (26%)	2 (4%)	14 (26%)
	Uttar Pradesh	161	156 (97%)	9 (6%)	147	131	4 (3%)	14 (11%)	37 (28%)	14 (11%)	18 (14%)	44 (34%)
Pakistan												
	Matiari	70	67 (96%)	0	67	63	0	17 (27%)	13 (21%)	8 (13%)	5 (8%)	20 (32%)
	Karachi	81	73 (90%)	2 (3%)	71	67	2 (3%)	11 (16%)	15 (22%)	9 (13%)	11 (16%)	19 (28%)
Democratic Republic of the Congo		75	50 (67%)	3 (6%)	47	46	6 (13%)	1 (2%)	18 (39%)	1 (2%)	6 (13%)	14 (30%)
Ghana		79	73 (92%)	2 (3%)	71	65	12 (18%)	10 (15%)	11 (17%)	8 (12%)	9 (14%)	15 (23%)
Kenya		29	29 (100%)	0	29	29	2 (7%)	5 (17%)	7 (24%)	9 (31%)	1 (3%)	5 (17%)
Tanzania												
	Ifakara	33	23 (70%)	0	23	21	1 (5%)	5 (24%)	5 (24%)	3 (14%)	2 (10%)	5 (24%)
	Pemba	66	61 (92%)	0	61	52	1 (2%)	12 (23%)	18 (35%)	6 (12%)	6 (12%)	9 (17%)
South Asia		508	445 (88%)	3% (1–7)†	427	384	2% (1–4)‡	21% (17–25)‡	23% (19–28)‡	14% (10–17)‡	12% (9–16)‡	27% (22–32)‡
Sub-Saharan Africa		282	236 (84%)	1% (0–4)†	231	213	9% (5–13)‡	15% (10–20)‡	29% (23–35)‡	12% (8–17)‡	12% (7–16)‡	23% (18–29)‡

Data are n, n (%), or % (95% CI).

* Zambia was excluded from calculation of cause of pregnancy-related death because of differences in the enrolment strategy of women of reproductive age.

† Proportion of maternal deaths based on total number column.

‡ Overall proportion (95% CI) was obtained from meta-analysis.

Verbal autopsies were done for 6549 (89%) of the 7335 stillbirths, with timing (antepartum or intrapartum) assigned for 6478 cases (88%), of which 3597 (56%) were antepartum and 2881 (44%) were intrapartum stillbirths (appendix). There were regional differences: during labour and up to 24 h after birth, the proportion of intrapartum stillbirths was just less than half in the south Asia sites (48%, 95% CI 39–57) but was 37% (95% CI 27–47) in the sub-Saharan Africa sites (figure 4; appendix). In sub-Saharan Africa, the proportion of intrapartum stillbirths was lowest in the two Tanzanian sites (approximately 20%).

A cause of death could be determined from the verbal autopsy in 2537 (71%) of the 3597 antepartum stillbirths (appendix) and in 2321 (81%) of the 2881 intrapartum stillbirths (appendix). Fetus-related causes were assigned for less than 10% of antepartum or intrapartum stillbirths. The major underlying causes of antepartum stillbirths were hypertensive disorders of pregnancy (south Asian sites 47%, 95% CI 45–49; sub-Saharan African sites 21%, 18–24), infections (south Asian sites 18%, 17–20; sub-Saharan African sites 50%, 46–53), and placental complications resulting in antepartum haemorrhage (south Asian sites 21%, 19–23; sub-Saharan African sites 15%, 13–18) (appendix). Together, these three conditions accounted for three-quarters of antepartum stillbirths for which the cause was known (figure 5C).

Complications of labour and delivery accounted for more than half of intrapartum stillbirths in south Asia sites and more than-two thirds of those in sub-Saharan Africa sites (appendix). The three other main causes were hypertensive disorders of pregnancy in south Asia, infections in sub-Saharan Africa, and antepartum haemorrhage. Together, these three conditions accounted for more than 80% of intrapartum stillbirths for which the cause was known (appendix; figure 5D).

Of 8244 neonatal deaths, timing of death was known for 7876 (96%) deaths and verbal autopsies were done for 7463 (91%) deaths. The highest burden occurred in the first 24 h after birth, both in the south Asian (44%, 95% CI 43–46) and in the sub-Saharan African sites (45%, 42–47; appendix). In Ifakara and Zambia, the proportion of deaths occurring in the first 24 h was lower than the rest of the sites (appendix). Approximately 20% of neonatal deaths occurred after the first week of life (figure 4; appendix).

A cause of death could be ascertained for 7066 (94·7%) of 7463 neonatal deaths for which a verbal autopsy was completed. The two most common causes of death were perinatal asphyxia (south Asian sites 40%, 95% CI 39–42; sub-Saharan African sites 34%, 32–36) and severe neonatal infections including sepsis, meningitis, and pneumonia (south Asian sites 35%, 34–36; sub-Saharan African sites 37%, 34–39; table 3). Complications of preterm birth accounted for about one in five neonatal deaths (south Asian sites 19%, 18–20; sub-Saharan African sites 24%, 22–26). Thus, these three conditions accounted for more than 90% of neonatal deaths (table 3; figure 5B).

**Table 3:** Causes of neonatal deaths, by site

		**Neonatal death**	**Verbal autopsy done**	**Cause determined**	**Preterm birth complications**	**Perinatal asphyxia**	Severe neonatal infection*	**Neonatal diarrhoea**	**Neonatal tetanus**	**Congenital malformation**	**Accident or injuries**	**Other perinatal causes**†
Bangladesh		995	971 (98%)	933	146 (16%)	342 (37%)	431 (46%)	0	4 (0%)	7 (1%)	1 (0%)	2 (0%)
India												
	Haryana	1399	1301 (93%)	1206	306 (25%)	475 (39%)	321 (27%)	5 (<1%)	2 (0%)	87 (7%)	4 (0%)	6 (0%)
	Uttar Pradesh	1575	1481 (94%)	1375	178 (13%)	531 (39%)	531 (39%)	11 (1%)	12 (1%)	54 (4%)	10 (1%)	48 (3%)
Pakistan												
	Matiari	1269	1221 (96%)	1167	226 (19%)	497 (43%)	377 (32%)	6 (1%)	2 (<1%)	42 (4%)	0	17 (1%)
	Karachi	862	795 (92%)	763	173 (23%)	307 (40%)	224 (29%)	8 (1%)	2 (<1%)	38 (5%)	3 (<1%)	8 (1%)
Democratic Republic of the Congo		173	108 (62%)	111	28 (25%)	23 (21%)	56 (50%)	0	1 (1%)	0	0	3 (3%)
Ghana		687	598 (87%)	578	169 (29%)	210 (36%)	161 (28%)	0	4 (1%)	16 (3%)	3 (1%)	15 (3%)
Kenya		397	271 (68%)	260	102 (39%)	82 (32%)	72 (28%)	1	0	3 (1%)	0	0
Tanzania												
	Ifakara	221	168 (76%)	159	22 (14%)	62 (39%)	72 (45%)	0	0	2 (1%)	0	1 (1%)
	Pemba	303	276 (91%)	253	33 (13%)	119 (47%)	79 (31%)	0	0	9 (4%)	1 (0%)	12 (5%)
Zambia		363	273 (75%)	261	72 (28%)	70 (27%)	97 (37%)	3 (1%)	2 (1%)	10 (4%)	1 (0%)	6 (2%)
South Asia		6100	5769 (95%)	5444	19% (18–20)‡	40% (39–42)‡	35% (34–36)‡	<1%	<1%	3% (3–4)‡	<1%	1% (1–2)‡
Sub-Saharan Africa		2144	1694 (79%)	1622	24% (22–26)‡	34% (32–36)‡	37% (34–39)‡	<1%	<1%	2% (2–3)‡	<1%	2% (2–3)‡

* Neonatal pneumonia, sepsis, or meningitis.

† Other perinatal cause or other conditions originating in the perinatal period.

‡ Overall proportion (95% CI) was obtained from meta-analysis.

When maternal deaths, stillbirths, and neonatal deaths are considered together, the period covering labour, birth, and the first 24 h after birth is the most critical for mortality prevention, followed by the third trimester of pregnancy and days 2–7 after birth (figure 4). Cause-specific maternal mortality ratios, stillbirth rates, and neonatal mortality rates are shown in the appendix.

## Discussion

To our knowledge, this is the largest study to provide population-based rates, timing, and causes of maternal deaths, stillbirths, and neonatal deaths from the same cohorts across 11 sites in eight countries of sub-Saharan Africa and south Asia, using harmonised methods. These two regions bear a substantial portion of the total global burden of maternal deaths, stillbirths, and neonatal deaths, but have the largest data gaps. The findings show that it is possible to generate harmonised, high-quality mortality data from these regions with ongoing research studies with other objectives, if additional resources are made available and an effective coordinating mechanism is established.

In our study, maternal mortality ratios were similar in both regions yet stillbirth rates and neonatal mortality rates were two times higher in south Asia sites compared with sub-Saharan Africa sites. A little less than half of the total number of pregnancy-related deaths, stillbirths, and neonatal deaths occurred during labour, delivery, and the subsequent 24 h, making it a key time to deliver interventions to improve survival. Obstetric haemorrhage, non-obstetric complications (indirect cause of maternal deaths), and hypertensive disorders in pregnancy accounted for more than two-thirds of maternal deaths. About two-thirds of antepartum stillbirths were caused by hypertensive disorders and infections during pregnancy. Approximately 70% of neonatal deaths were caused by perinatal asphyxia and neonatal infections, highlighting the need to develop effective, preventive antenatal and intrapartum care interventions.

We prospectively followed cohorts of pregnant women and produced high-quality data from the two regions with the highest burden with the least availability of accurate data. The study had a large sample size, allowing high precision, and sites were spread across the west, north, and east of south Asia, and the west, centre, and east of Africa, which makes the findings generalisable to these global regions.

Unlike most available data that are based on pregnancy history obtained at cross-sectional surveys and which are prone to selection and recall biases, our study was a population-based cohort study in which more than 95% of all reported pregnancies were followed to the end of the postpartum period. Thus, our study is less likely to have recall and reporting biases.

A further strength of this design is that pregnancy-related deaths, newborn deaths, and stillbirths were studied together in the same cohort, allowing us to reliably study their inter-relationships. This is the first large population-based study to report causes of stillbirths. Data collection lasted at least two full calendar years in all sites, which mitigates the effect of seasonal variations in mortality and its causes.

Finally, a high level of harmonisation was achieved across sites in the design, implementation, and analysis of data through use of harmonised data collection tools, common standard operating procedures, training, standardisation of determination of causes of death, and quality assurance. In particular, standardisation of assignment of causes of death by physicians has been particularly inadequate in previous studies.

Regarding limitations, there were only one or two cohorts per country, and although these were large study populations, they are unlikely to be representative of the entire country, particularly in large countries such as India and the Democratic Republic of the Congo. There were large variations across sites, which may have resulted from selection of a certain type of site population in a country. For example, Kenya was consistently the outlier and was at the lower end for all mortality rates. There were also some variations in sites in the same country (ie, India, Pakistan, and Tanzania). However, some of these variations are explained by the contextual differences in the study sites and are reflected in the wide confidence intervals for mortality rates pooled for south Asia and sub-Saharan Africa (appendix).

Second, it is likely that we have missed early abortions (spontaneous or induced), particularly those in the first trimester, because of our routine 2–3 monthly surveillance, and because women might not have declared themselves to be pregnant until well into the second trimester. Furthermore, abortion is likely to be under-reported because of legal or cultural reasons. We conducted verbal autopsies for all deaths in women of reproductive age, and not just those who identified themselves to us as being pregnant, to reduce underestimation of abortion as a cause of maternal mortality, but the respondent might not have known about the pregnancy or the abortion.

Third, the routine follow-up of study participants in the context of the original studies might have resulted in earlier recognition of maternal or newborn illness and referral for care, particularly related to the participant of the original study.^11^ This might have had an effect on mortality rates or some of the causes of death, making them appear lower than what might have been the case in these communities in the absence of the study-related activities. However, the mortality rates were high in most sites. Furthermore, although many of the original studies focused on newborn infections, we still found that newborn infections were one of the most common causes of neonatal deaths.

Finally, even though we did verbal autopsies in the best possible manner, their inherent problems for determining the causes of death must be acknowledged when interpreting the results. Despite its limitations, verbal autopsy remains the only feasible method for ascertaining causes of death in study populations in which a large proportion of deaths occur at home and health facility records are often inadequate. Final assignment of causes of death was based on agreement between physician coders. Although there have been attempts in recent years to develop computer-based methods to analyse verbal autopsies, an approach that we had initially planned to undertake, we decided against this approach because these methods have not been proven to be better than well conducted assignment by unbiased physicians.^21^

By comparison with previously published results, there are two published estimates of maternal mortality ratio that show different results; one suggests that maternal mortality in sub-Saharan Africa is much higher than in south Asia,^1^ and the other indicates that the two regions have almost the same pooled estimate, which is consistent with our findings.^22^ Previously published estimates of stillbirth rates and neonatal mortality rates show that the rates are similar in the two regions or slightly higher in sub-Saharan Africa.^2^, ^3^, ^23^, ^24^ Our results indicate that both stillbirth rates and neonatal mortality rates are roughly two times higher in south Asia than in sub-Saharan Africa. As discussed previously, this difference could be due to selection of sites in south Asia where the socioeconomic and health systems contexts were poorer than the regional average, and selection of sites in sub-Saharan Africa where these contexts were better than the regional average. However, our finding that maternal mortality was similar in sites in the two regions indicates that the differences in stillbirth rate and neonatal mortality rate might not be explained by context. Other possible explanations could be greater incidence of low birthweight, poorer maternal nutrition and smaller height, and lower societal status of women in south Asia compared with sub-Saharan Africa.

Estimates from the Global Burden of Disease study somewhat surprisingly show that in 2013, on average, nearly 25% of maternal deaths occurred antepartum, 25% occurred intrapartum and immediately postpartum, more than 33% between 2 and 42 days after birth, and the remaining deaths between 42 days and 1 year after birth.^22^ This is by contrast with the conclusion in the *Lancet* Maternal Survival Series^25^ that the risk of maternal death is at maximum intrapartum and the 24 h after birth. Our findings show that the largest proportion of maternal deaths occur in the intrapartum period and the first 24 h after birth, followed by the postpartum period, and then the antepartum period, which are consistent with conclusions of the *Lancet* Maternal Survival Series.

Previous estimates of stillbirth rates suggest that more than half of stillbirths in south Asia and sub-Saharan Africa are intrapartum.^26^ However, we found that more than half of stillbirths were antepartum. These differences could be because of more complete pregnancy data in our cohorts, compared with data sources for the estimates.

It has been reported that more than a third of neonatal deaths in south Asia and sub-Saharan Africa occur on the first day of life.^27^ We found that an even higher proportion of deaths occur on the first day, accounting for almost half of all neonatal deaths. It is noteworthy that this proportion was equally high in south Asia, despite a two-times higher neonatal mortality rate than that in sub-Saharan Africa. The possible reasons for difference in results could be older source data for estimates, as well as reporting or misclassification problems in those data.

Previously published estimates indicate that indirect medical causes, haemorrhage, hypertension, sepsis, and abortion are the five most important causes of maternal deaths in sub-Saharan Africa and south Asia.^6^ Another published paper reported a lower proportion of indirect causes and a higher proportion due to direct causes, along with haemorrhage, hypertension sepsis, and abortion as the most common causes of maternal deaths.^22^ Our findings were similar to the former, the only exception being that we found a lower proportion of abortion deaths in our study. As noted previously, we are likely to have missed abortion deaths because of the way we assembled our cohorts.

We did not find any previously published estimates of causes of stillbirth in south Asia and sub-Saharan Africa.

Published estimates of causes of newborn deaths over the past 15 years have shown that newborn deaths due to infections and perinatal asphyxia have been decreasing and those due to preterm birth complications have been increasing. The most recent estimates show that preterm birth is the most common cause of death in all regions of the world, particularly in south Asia.^5^ Our findings are quite different. In both south Asia and sub-Saharan Africa, perinatal asphyxia and newborn infections are the first and second causes of neonatal death, respectively, followed by preterm birth complications. These differences could be because of incorrect assignment of preterm birth as the cause of death for many babies who are born preterm but do not die because of preterm birth complications in input data used for global and regional estimates.

Our findings and the data available from this study can help improve the global modelled estimates in two ways. First, the addition of our data will increase the quality of input data available for modelling. Second, the models, which are dependent on covariate factors because mortality data are scarce or unavailable, can themselves be validated and improved by examining how they predict mortality within our datasets. Additionally, our findings will be helpful in subnational settings for refining health programmes and planning at local level. Needless to say, establishing vital registration systems and improving quality, frequency, and sampling locations for primary data collection will be essential to improve the availability of data to monitor the SDG targets.

Our study also has several implications for public health programmes in both south Asia and sub-Saharan Africa. First, our findings suggest that stillbirths and neonatal mortality rates in south Asia might be higher than those suggested by surveys; thus, programmes need to further intensify delivery of effective interventions to reduce stillbirths and newborn deaths. In particular, there is limited focus on stillbirths in global accountability and SDG monitoring.

Second, nearly half of the stillbirths and neonatal deaths occurred during labour and on the day of birth, which means the focus on improving the quality of maternal intrapartum care and immediate newborn care must be further enhanced. The causes of stillbirth confirm the large overlap and synergy with efforts to improve maternal survival.

Third, more than half of stillbirths occurred in the antepartum period, and the implementation of the new WHO antenatal care model^28^ with intensification of the frequency of antenatal visits in the last trimester of pregnancy might help reduce this burden.

Fourth, about a third of maternal mortality occurs beyond 24 h of birth, and therefore better interventions to prevent, detect, and manage complications in the postpartum period need to be developed and tested.

Fifth, the absence of a clear relationship between facility births and stillbirths or neonatal mortality in populations implies that promoting facility births, without addressing what is necessary to improve quality of care, might be inappropriate. This finding emphasises the need to improve quality of care in health facilities. In particular, we strongly believe that achieving high-quality intrapartum and postnatal care at health facilities is the missing complementary piece required to improve survival.

Finally, the current regional and country estimates could be giving policy makers and programme managers in south Asian and sub-Saharan African countries a false sense that newborn infections and perinatal asphyxia are no longer the most important causes to address, and over-emphasising the importance of preterm birth complications. Our findings imply that it is crucial to address all three of these causes of death to achieve survival goals in the SDG era.

Correspondence to: Dr Rajiv Bahl, Department of Maternal, Newborn, Child and Adolescent Health, World Health Organization, Geneva 1211, Switzerland bahlr@who.int

or

Dr Sachiyo Yoshida, Department of Maternal, Newborn, Child and Adolescent Health, World Health Organization, Geneva 1211, Switzerland yoshidas@who.int
