# Supplementary material for: Population-based rates, timing, and causes of maternal deaths, stillbirths, and neonatal deaths in south Asia and sub-Saharan Africa: a multi-country prospective cohort study
Source: Lancet Glob Health. 2018 Oct 22;6(12):e1297–308. doi: 10.1016/S2214-109X(18)30385-1 (PMC6227247; doi:10.1016/S2214-109X(18)30385-1)
Supplement: Supplementary appendix [file mmc1.pdf]

THE LANCET
Global Health

Supplementary appendix

This appendix formed part of the original submission and has been peer reviewed. We post it as supplied by the authors.

Supplement to: The Alliance for Maternal and Newborn Health Improvement (AMANHI) mortality study group. Population-based rates, timing, and causes of maternal deaths, stillbirths, and neonatal deaths in south Asia and sub-Saharan Africa: a multi-country prospective cohort study. *Lancet Glob Health* 2018; published online Oct 22. http://dx.doi.org/10.1016/S2214-109X(18)30385-1.

REVISED

Dated 11 October 2018

Web appendix

**Web table 1.**	(a) Summary description of the parent study, site setting, surveillance population and annual number of births, and (b) survey-based estimates of MMR, NMR, ANC coverage & SBA coverage in the regions in which the studies were conducted, compared with national estimate
**Web table 2.**	(a) Event definitions and (b) definitions of underlying causes of death
**Web table 3.**	Timing of (a) pregnancy related deaths by site and region, (b) stillbirths by site and region and (c) neonatal deaths by site and region
**Web table 4.**	Causes of antepartum stillbirths by site
**Web table 5.**	Causes of intrapartum stillbirths by site
**Web table 6.**	Cause-specific (a) maternal mortality ratios, (b) stillbirth rates and (c) neonatal mortality rates
**Web Figure 1.**	Meta-analysis forest plots for: (i) Maternal Mortality Ratios; (ii) Stillbirth Rates; and (iii) Neonatal Mortality Rates
**Web Figure 2.**	Timing of pregnancy related deaths, stillbirth and neonatal deaths in each region

In case they need to be removed from the main paper

**Web Figure 3.**	Relationships of (i) stillbirth rates (ii) neonatal deaths with percentage of births in health facilities
**Web Figure 4.**	Relationships of (i) stillbirth rates and (ii) neonatal mortality rates with percentage of mothers with any schooling

Web table 1a. Summary description of the parent study, site setting, surveillance population and annual number of births

Country (Region in which the study was conducted)	Parent study title and objective	Setting	Pregnancy Surveillance System[1]	Total Surveillance Population	Reproductive age women in Surveillance	Approximate annual births
Bangladesh (Sylhet)	Aetiology of Neonatal Infection in South Asia (ANISA): To estimate community level, aetiology-specific incidence of serious infections among infants (0-59 days). *Screening and Treatment of Maternal Genitourinary Tract Infections in early Pregnancy to Prevent Preterm Birth in Rural Sylhet, Bangladesh: A cluster randomized trial*	Rural areas of Sylhet district (Projahnmo trial area)	2-monthly by trained community health workers (CHWs)	500,000	88,000	12,500
DR Congo (North and South Ubangi)	African Neonatal Sepsis Trial (AFRINEST): To test safety and efficacy of simplified antibiotic regimens for treating possible bacterial infection in infants (0-59 days).	Rural area in the province of Equateur	3-monthly CHWs	699,288	65,000	12000
Ghana (Brong Ahafo)	Neonatal vitamin A supplementation (NEOVITA) study: To assess the efficacy of neonatal vitamin A supplementation on neonatal and infant mortality.	Rural areas in the central Ghana	Monthly fieldworkers	700,000	147,000	21000
India (Haryana)	Neonatal vitamin A supplementation (NEOVITA) study: To assess the efficacy of neonatal vitamin A supplementation on neonatal and infant mortality.	Faridabad and Palwal districts in the state of Haryana including 3 urban slum clusters in Faridabad district	Monthly by CHWs	1,400,000	313,399	34600
India (UP)	AMANHI Study (parent study delayed)	Rural area in Uttar Pradesh	3-monthly by FWs	1,350,000	184,430	44000
Kenya (Western)	African Neonatal Sepsis Trial (AFRINEST): To test safety and efficacy of simplified antibiotic regimens for treating possible bacterial infection in infants (0-59 days).	Mainly rural multi-ethnic area in western province	3-monthly by CHWs	400,000	30,000	10000
Pakistan (Karachi)	Aetiology of Neonatal Infection in South Asia (ANISA): To estimate community level, aetiology-specific incidence of serious infections among infants (0-59 days).	4 peri-urban communities in Bin Qasim town and 1 urban low-income community in Korangi town	3-monthly by FWs	270,000	63,000	9500

Pakistan (Matiari)	Aetiology of Neonatal Infection in South Asia (ANISA): To estimate community level, aetiology-specific incidence of serious infections among infants (0-59 days).	Rural area in Sindh district	3-monthly by FWs	215,200	64,000	8000
Tanzania (Ifakara)	Neonatal vitamin A supplementation (NEOVITA) study: To assess the efficacy of neonatal vitamin A supplementation on neonatal and infant mortality.	Ifakara town and surrounding rural Kilombero, Ulanga and Kilosa districts in Morogoro region	3-monthly by FWs	300,000	72,000	6000
Tanzania (Pemba)	Chlorhexidine (CHX) study: To evaluate the efficacy of chlorhexidine cord cleansing on neonatal mortality	Pemba Island in the Zanzibar archipelago	6-weekly by CHWs	390,000	72,000	14000
Zambia (Southern)	Chlorhexidine (CHX) study: To evaluate the efficacy of chlorhexidine cord cleansing on neonatal mortality	Six rural districts in Southern Province	No pregnancy surveillance; facility ANC enrolment	25,000	25,000	9000

1. Frequency of home-visits and staff responsible for home-visits.

Web table 1b. Survey-based estimates of MMR, NMR, ANC coverage & SBA coverage in the regions in which the studies were conducted, compared with national estimate

Country (Region in which the study was conducted)	Maternal mortality **National** (per 100 000 lbs)[1]	Neonatal mortality (per 1000 lbs)[2]		ANC visit (≥1)[3]		Skilled birth attendant at birth[3]	
		**National estimate**	**Regional estimate**	**National estimates**	**Regional estimates**	**National estimates**	**Regional estimates**
Bangladesh (Sylhet)	176 [125-280]	28	39	79%	62%	42%	27%
DR Congo (North and South Ubangi)	693 [509-1010]	28	28	98%	87%	80%	64%
Ghana (Brong Ahafo)	319 [216-458]	27	27	97%	99%	74%	79%
India (Haryana)	174 [139-217]	28	26	74%	Not available	76%	85%
India (UP)	174 [139-217]	28	35	74%	Not available	76%	63%
Kenya (Western)	510 [344-754]	22	19	96%	98%	62%	48%
Pakistan (Karachi)	178 [111-283]	55	42	76%	79%	52%	61%
Pakistan (Matiari)	178 [111-283]	55	62	76%	79%	52%	61%
Tanzania (Ifakara)	398 [281-570]	26	38	98%	99%	51%	76%
Tanzania (Pemba)	398 [281-570]	26	25	98%	99%	51%	34%
Zambia (Southern)	224 [162-306]	24	23	98%	99%	64%	55%

1. WHO, UNICEF, UNFPA, World Bank Group and the United Nations Population Division. Trends in maternal mortality: 1990 to 2015
Estimates by WHO, UNICEF, UNFPA, World Bank Group and the United Nations Population Division. WHO Library Cataloguing-in-Publication Data. http://apps.who.int/iris/bitstream/10665/194254/1/9789241565141_eng.pdf?ua=1. Regional estimates of MMR not available.

2. The data presented is at regional/provincial/zone level where the study was conducted. Source: DHS for Sylhet division in Bangladesh (2014), Equateur province in DR Congo (2014), Brong Ahafo region in Ghana (2014), Western region in Kenya (2014), Sindh region in Pakistan (2012-2013), Eastern zone and Pemba island in Tanzania (2011) and Southern region in Zambia (2014). Data on Haryana and Uttar Pradesh is sourced from Sample Registration System India (2013).

3. The data presented is at regional/provincial/zone level where the study was conducted. Source: DHS for Sylhet division in Bangladesh (2014), Equateur province in DR Congo (2014), Brong Ahafo region in Ghana (2014), Western region in Kenya (2014), Sindh region in Pakistan (2012-2013), Eastern zone and Pemba island in Tanzania (2011) and Southern region in Zambia (2014). Data on Haryana and Uttar Pradesh, India is sourced from National Family Health survey-4 (2015-2016) and Annual Health Survey (2012-2013) respectively. Both data accessed on 15 June 2016. For the national level data on antenatal care and skilled birth attendant, the data are from State of India's Newborn 2014 and State of World Children 2015 (both accessed 25th July, 2016).

Web table 2a. **Event definitions**

Event Definitions	Pregnancy-Related Death	Death of a woman while pregnant or within 42 days of termination of pregnancy, regardless of cause of death
	Maternal Death	Pregnancy-related death from any cause related to or aggravated by the pregnancy or its management, but not from accidental or incidental causes
	Neonatal Death	Death of a liveborn baby within 28 days of birth
	Antepartum Stillbirth	Foetal death occurring after 28 weeks of gestation and before the onset of labour
	Intrapartum Stillbirth	Foetal deaths occurring after the onset of labour and before the delivery
Summary Measures	Maternal Mortality Ratio (MMR)	Number of maternal deaths per 100 000 live births
	Neonatal Mortality Rate (NMR)	Number of neonatal deaths per 1000 livebirths
	Stillbirth Rate (SBR)	Number of stillbirths per 1000 births

Web table 2b. **Definitions of underlying causes of: (a) pregnancy related deaths; (b) stillbirths; and (c) neonatal deaths**

(a) Underlying causes of pregnancy related deaths	Directions for physicians in assigning the causes of death
Pregnancy with abortive outcomes	Abortion, miscarriage, ectopic pregnancy leading to maternal death within 42 days of the abortive outcome<br><br>a) Spontaneous or induced abortions<br>• Bleeding usually followed by pain<br>• History of passing products of pregnancy per vaginum<br>• May only present with complications such as<br>– Continued bleeding<br>– Ruptured/perforated uterus following instrumentation.<br>– Symptoms of sepsis: foul -smelling discharge, fever, organ failure.<br>b) Ectopic pregnancy:<br><br>• Usually in the 1st trimester<br>• Severe, sharp abdominal pain, may be followed by bleeding<br>• May be associated with:<br>– Unconsciousness<br>– Abdominal distension<br>– Shock
Hypertensive disorders in pregnancy, childbirth and puerperium	Pre-eclampsia and eclampsia:<br><br>• Health worker told the mother she had high BP<br>• Puffiness of the face, headaches and blurring of vision<br>• Presence of symptoms of complications: convulsions, renal failure, pulmonary edema, cerebro -vascular accidents

Obstetric haemorrhage	Obstetric disease or conditions directly associated with hemorrhage:<br><br>Bleeding before onset of labour.<br><br>**Abruptio**<br><br>• Severe abdominal pain (constant or intermittent)<br>• Uterine tenderness<br>• Vaginal bleeding (may be absent)<br>**Praevia**<br><br>• Vaginal bleeding without pain<br>Bleeding during and after the delivery:<br><br>• Excessive bleeding during or after delivery<br>• May be associated with difficulty in delivering the placenta or incomplete delivery of placenta<br>• May be associated with shock
Pregnancy -related infection	Antepartum infection:<br><br>Symptoms appeared before onset of labour<br><br>• Fever, dysuria, abdominal or loin pain, haematuria, foul -smelling discharge, etc.<br>• Shock<br>Postpartum infection:<br><br>Symptoms appear after the birth of the baby<br><br>• Fever, foul smelling or purulent vaginal discharge, lower abdominal pain, dysuria, loin pain, haematuria, etc.<br>• Shock
Other obstetric complications	Direct obstetric conditions not included in all other causes:<br><br>• Labour lasting more than 24 hours<br>• Difficult or complicated delivery<br>• May be associated with abnormal position of the baby (transverse or oblique lie)

	• Ruptured uterus (severe abdominal pain, shock, health worker told the family that the uterus had ruptured)
Non -obstetric complications	Woman who is pregnant and has a pre -existing medical condition that can explain the circumstances leading to the death should be **assumed** to have a medical condition exacerbated by the pregnancy.<br>a. Rheumatic heart disease,<br>b. hypertension,<br>c. diabetes, etc.<br>d. Severe anaemia (a health worker told the mother that she had very low haemoglobin, history of pallor with symptoms such as lethargy and easy fatigability, difficulty in breathing, swelling of the legs, upper abdominal tenderness and prominent neck veins)
Coincidental causes	Fall, road traffic accident, burns, poisoning, drowning, animal bite<br>• Severe enough to cause death<br>• Temporal, direct relationship with events that lead to death

**(b) Underlying causes of stillbirths**	**Directions for physicians in assigning the causes of death**
Congenital malformations	Potentially lethal malformations including head not formed, very large head, large defect in the back of head or in the spine
Other specific fetal cause	Any specific fetal cause of death other than congenital malformations
Maternal medical conditions:<br><br>Other medical conditions	Rheumatic heart disease, hypertension, diabetes, epilepsy, etc.
Maternal medical conditions: Hypertensive disorder	Conditions including the following:<br><br>• Health worker told the mother she had high BP<br>• Puffiness of the face, headaches and blurring of vision<br>• Presence of symptoms of complications: convulsions, renal failure, pulmonary edema, cerebro -vascular accidents
Maternal medical conditions: infections	History of chorio -amnionitis or TORCH infections or severe malaria
Maternal medical conditions: Accidents or injuries	• Fall, road traffic accident, burns, poisoning, drowning, animal bite , etc.<br>• Severe enough to cause stillbirth<br>• Temporal, direct relationship with events that lead to stillbirth
Complication of placenta: Antepartum haemorrhage	Bleeding before onset of labour which includes the following:<br><br><u>Abruptio</u><br><br>• Severe abdominal pain (constant or intermittent)<br>• Uterine tenderness<br>• Vaginal bleeding (may be absent)<br><u>Praevia</u><br><br>• Vaginal bleeding without pain
Complications of labour and	Conditions including the following:

delivery	• Labour lasting more than 24 hours<br>• Difficult or complicated delivery<br>• Abnormal position of the baby (transverse or oblique lie)<br>• Umbilical cord prolapse<br>• Malpresentations: breech, feet or hand delivered first

(c) **Underlying causes of neonatal deaths**	**Directions for physicians in assigning the causes of death**
Preterm birth complications	Gestation ≤7 months and died within 4 hours of birth<br><br>Gestation ≤8 months and had:<br><br>– Breathing difficulty starting within 4 hours of birth<br>– Neurological signs<br>– Bleeding from nose and mouth<br>– Abdominal distention after starting feeding
Perinatal asphyxia	Baby born at ≥8 months gestation:<br><br>▪ Did not cry or breathe within 5 minutes of birth, or breathed only with assistance (such as bag and mask ventilation)<br>▪ No other signs required if death within 6 hours of birth<br>▪ If death after 6 hours of birth, had neurological signs (irritability, convulsions, lethargy or unconsciousness)<br>▪ Baby not normal any time after birth
Severe neonatal infection	Neonatal sepsis or meningitis including any of the following symptoms appearing on day 3 or later, with or without respiratory symptoms:<br><br>• Stopped feeding well<br>• Fever<br>• Cold to touch<br>• Lethargy<br>• Convulsions<br>Any of the above symptoms appearing on day 1 or 2, and PROM >18 h, foul -smelling liquor or fever<br><br>Baby was well for some time after birth<br><br>Neonatal pneumoniaincluding following symptoms:<br><br>• Baby was normal in the first 2 days after birth, symptoms largely respiratory, appearing on day 3 of life or later including:

	• Fast breathing, or<br>• Difficulty in breathing, or<br>• Chest indrawing<br>• May have developed generalized symptoms of infection close to death.
Neonatal diarrhoea	The only major symptom is diarrhoea<br><br>Profuse watery and frequent stools
Neonatal tetanus	Baby with normal feeding and activity in the first two days after birth, and then developed<br><br>▪ locked jaw and inability to feed, and<br>▪ Arched back, or spasms
Congenital malformation	Potentially lethal malformations e.g.<br><br>• Head not formed, very large head, large defect in the back of head or in the spine<br>• Blue lips and tongue, or a health worker informed family that baby has a severe heart malformation<br>• Frothing from the mouth associated with choking, coughing, vomiting, and becoming blue after feeding was started<br>• Abdominal wall not formed, no anal opening, or severe abdominal distention and vomiting in the first days of life
Accidents/injuries	Accident or injury occurred after birth (fall, road traffic accident, burns, poisoning, drowning, animal bite)<br>- Severe enough to cause death<br>- Temporal, direct relationship with events that lead to death<br>Does not include birth injury
Other perinatal causes	A specific cause of death which is not listed among the above -mentioned underlying causes of deaths

Web table 3a: Timing of Pregnancy Related Deaths by Site and Region

SITE	Pregnancy related death	Timing known	1st Trimester	2nd Trimester	3rd Trimester	Labor and birth up to 24 hours	Day 2-7	Day 8-42
	N	N	N (%)	N (%)	N(%)	N(%)	N(%)	N(%)
BAN	122	**121**	4 (3%)	1 (1%)	13 (11%)	48 (40%)	17 (14%)	38 (31%)
IND (H)	74	**69**	2 (3%)	11 (16%)	15 (22%)	17 (25%)	10 (14%)	14 (20%)
IND (U)	161	**151**	4 (3%)	6 (4%)	28 (19%)	75 (50%)	17 (11%)	21 (14%)
PAK (M)	70	**67**	0 (0%)	5 (7%)	11 (16%)	33 (49%)	9 (13%)	9 (13%)
PAK (K)	81	**76**	2 (3%)	6 (8%)	5 (7%)	41 (54%)	7 (9%)	15 (20%)
DRC	75	**52**	4 (8%)	10 (19%)	14 (27%)	19 (37%)	2 (4%)	3 (6%)
GHA	79	**75**	13 (17%)	9 (12%)	11 (15%)	20 (27%)	6 (8%)	16 (21%)
KEN	29	**24**	1 (4%)	2 (8%)	4 (17%)	8 (33%)	0 (0%)	9 (38%)
TAN (I)	33	**25**	0 (0%)	1 (4%)	0 (0%)	11 (44%)	7 (28%)	6 (24%)
TAN (P)	66	**65**	3 (5%)	1 (2%)	8 (12%)	32 (49%)	7 (11%)	14 (22%)
SA	508	484	3% (1-4)	6% (4-8)	15% (12-18)	44% (40-49)	13% (10-16)	20% (17-24)
SSA	282	241	7% (4-10)	8% (5-12)	15% (10-19)	40% (34-46)	9% (5-13)	21% (16-27)

Web table 3b: Timing of Stillbirths by Site and Region

SITE	SB	SB Timing known	Antepartum	Intrapartum
	N	N	N (%)	N (%)
BAN	1068	**931**	392 (42%)	539 (58%)
IND (H)	798	**757**	293 (39%)	464 (61%)
IND (U)	1479	**1382**	786 (57%)	596 (43%)
PAK (M)	1211	**1115**	654 (59%)	461 (41%)
PAK (K)	675	**605**	380 (63%)	225 (37%)
DRC	155	**124**	63 (51%)	61 (49%)
GHA	654	**623**	398 (64%)	225 (36%)
KEN	233	**147**	68 (46%)	79 (54%)
TAN (I)	123	**60**	48 (80%)	12 (20%)
TAN (P)	498	**430**	340 (79%)	90 (21%)
ZAM	441	**304**	175 (58%)	129 (42%)
SA	5231	**4790**	52% (43 - 61)	48% (39 - 57)
SSA	2104	**1688**	63% (53 - 73)	37% (27 - 47)

Web table 3c: Timing of Neonatal Deaths by Site and Region

SITE	Neonatal death	Timing known	Day 1(Birth to 24 hours)	Day 2	Day 3	Day 4 - Day 7	Day 8- Day 28
	N	N	N (%)	N (%)	N (%)	N (%)	N (%)
BAN	995	**995**	420 (42%)	158 (16%)	91 (9%)	111 (11%)	215 (22%)
IND (H)	1399	**1287**	536 (42%)	140 (11%)	107 (8%)	165 (13%)	339 (26%)
IND (U)	1575	**1575**	822 (52%)	91 (6%)	111 (7%)	270 (17%)	281 (18%)
PAK (M)	1269	**1198**	497 (41%)	147 (12%)	112 (9%)	179 (15%)	263 (22%)
PAK (K)	862	**803**	337 (42%)	86 (11%)	74 (9%)	125 (16%)	181 (23%)
DRC	173	**147**	80 (54%)	15(10%)	12 (8%)	17 (12%)	23 (16%)
GHA	687	**681**	308 (45%)	44 (6%)	52 (8%)	115 (17%)	162 (24%)
KEN	397	**359**	172 (48%)	54 (15%)	30 (8%)	31 (9%)	72 (20%)
TAN (I)	221	**218**	74 (34%)	39 (18%)	39 (18%)	33 (15%)	33 (15%)
TAN (P)	303	**259**	131 (51%)	39 (15%)	26 (10%)	28 (11%)	35 (14%)
ZAM	363	**354**	124 (35%)	69 (19%)	33 (9%)	53 (15%)	75 (21%)
SA	6100	5858	44% (43-46)	11% (10-12)	9% (8-9)	14% (13-15)	22% (21-23)
SSA	2144	2018	45% (42-47)	13% (12-15)	10% (9-11)	13% (12-15)	19% (17-21)

Web table 4: Causes of antepartum stillbirths by site

SITE	Number[2]	Cause determined	Congenital malformations	Other specific fetal cause	Maternal medical conditions: Hypertensive disorder of pregnancy	Infections	Other medical conditions	Accident/Injury	Complications of placenta: Antepartum Haemorrhage	Complications of labour and delivery
	N	N	N (%)	N (%)	N (%)	N (%)	N (%)	N (%)	N (%)	N (%)
BAN	392	**303**	0 (0%)	0 (0%)	135 (45%)	23 (8%)	4 (1%)	0 (0%)	28 (9%)	113 (37%)
IND (H)	293	**180**	21 (12%)	23 (13%)	50 (28%)	43 (24%)	4 (2%)	7 (4%)	32 (18%)	0 (0%)
IND (U)	786	**623**	20 (3%)	76 (12%)	72 (12%)	143 (23%)	79 (13%)	24 (4%)	171 (27%)	38 (6%)
PAK (M)	654	**545**	23 (4%)	3 (1%)	390 (72%)	49 (9%)	7 (1%)	4 (1%)	69 (13%)	0 (0%)
PAK (K)	380	**228**	17 (7%)	9 (4%)	126 (55%)	20 (9%)	1 (0%)	9 (4%)	45 (20%)	1 (0%)
DRC	63	**55**	0 (0%)	0 (0%)	4 (7%)	39 (71%)	1 (2%)	0 (0%)	7 (13%)	4 (7%)
GHA	398	**230**	4 (2%)	11 (5%)	30 (13%)	93 (40%)	34 (15%)	1 (0%)	52 (23%)	5 (2%)
KEN	68	**34**	3 (9%)	0 (0%)	4 (12%)	19 (56%)	1 (3%)	0 (0%)	4 (12%)	3 (9%)
TAN (I)	48	**39**	1 (3%)	0 (0%)	14 (36%)	16 (41%)	1 (3%)	0 (0%)	7 (18%)	0 (0%)
TAN (P)	340	**182**	3 (2%)	2 (1%)	82 (45%)	36 (20%)	21 (12%)	0 (0%)	38 (21%)	0 (0%)
ZAM	175	**118**	5 (4%)	20 (17%)	18 (15%)	55 (47%)	1 (1%)	0 (0%)	4 (3%)	15 (13%)
**SA**	**2505**	**1879**	4% (3-5)[1]	3% (2-4)[1]	47% (45-49)[1]	18% (17-20)[1]	2% (2-3)[1]	2% (1-2)[1]	21% (19-23)[1]	3% (2-4)[1]
**SSA**	**1092**	**658**	3% (2-4)[1]	2% (1-3)[1]	21% (18-24)[1]	50% (46-53)[1]	4% (3-6)[1]	<1% (0-0.5)[1]	15% (13-18)[1]	5% (3-7)[1]

1. Overall % (CI) was obtained from meta-analysis
2. Type of stillbirth determined by VA

Web table 5: Causes of intrapartum stillbirths by site

SITE	Number[2]	Cause determined	Congenital malformations	Other specific fetal cause	Maternal medical conditions: Hypertensive disorder of pregnancy	Maternal medical conditions: Infections	Maternal medical conditions: Other medical conditions	Maternal medical conditions: Accident/Injury	Complications of placenta: Antepartum Haemorrhage	Complications of labour and delivery
	N	N	N (%)	N (%)	N (%)	N (%)	N (%)	N (%)	N (%)	N (%)
BAN	539	**452**	2 (0%)	0 (0%)	109 (24%)	11 (2%)	7 (2%)	0 (0%)	18 (4%)	305 (67%)
IND (H)	464	**329**	42 (13%)	24 (7%)	39 (12%)	37 (11%)	4 (1%)	0 (0%)	45 (14%)	138 (42%)
IND (U)	596	**509**	9 (2%)	100 (20%)	27 (5%)	51 (10%)	31 (6%)	3 (1%)	49 (10%)	239 (47%)
PAK (M)	461	**374**	10 (3%)	6 (2%)	123 (33%)	19 (5%)	2 (1%)	1 (0%)	37 (10%)	176 (47%)
PAK (K)	225	**166**	2 (1%)	12 (7%)	47 (28%)	6 (4%)	0 (0%)	3 (2%)	16 (10%)	80 (48%)
DRC	61	**59**	0 (0%)	1 (2%)	0 (0%)	9 (15%)	0 (0%)	0 (0%)	5 (8%)	44 (75%)
GHA	225	**191**	2 (1%)	2 (1%)	6 (3%)	22 (12%)	3 (2%)	0 (0%)	22 (12%)	134 (70%)
KEN	79	**62**	2 (3%)	4 (6%)	4 (6%)	8 (13%)	0 (0%)	0 (0%)	10 (16%)	34 (55%)
TAN (I)	12	**10**	0 (0%)	0 (0%)	3 (30%)	2 (20%)	0 (0%)	0 (0%)	1 (10%)	4 (40%)
TAN (P)	90	**70**	1 (1%)	1 (1%)	5 (7%)	3 (4%)	0 (0%)	0 (0%)	14 (20%)	46 (66%)
ZAM	129	**99**	1 (1%)	14 (14%)	2 (2%)	7 (7%)	0 (0%)	0 (0%)	6 (6%)	69 (70%)
**SA**	**2285**	**1830**	2% (2-3)[1]	3% (2-4)[1]	20% (18-21)[1]	6% (5-7)[1]	1% (1-2)[1]	<1% [1]	10% (9-11)[1]	57% (55-59)[1]
**SSA**	**596**	**491**	1% (0-2)[1]	3% (2-5)[1]	4% (3-6)[1]	11% (8-13)[1]	<1% [1]	0%	12% (9-15)[1]	69% (64-73)[1]

1. Overall % (CI) was obtained from meta-analysis
2. Type of stillbirth determined by VA

Web table 6a: Cause-specific maternal mortality ratio (per 100 000 livebirths)

Number				MMR per 100 000 livebirths						
SITE	Pregnancy Related Death	Maternal Death	Livebirths	Overall	Pregnancy with abortive outcomes	Hypertensive disorders in pregnancy, childbirth and puerperium	Obstetric haemorrhage	Pregnancy related infection	Other obstetric complications	Non-obstetric complications
BAN	122	120	26,295	456	13	150	105	39	98	52
IND (H)	74	67	35,000	191	4	47	33	51	7	51
IND (U)	161	151	37,813	399	12	43	113	43	55	134
PAK (M)	70	70	27,062	259	0	70	53	33	20	82
PAK (K)	81	79	17,189	460	14	75	103	62	75	131
DRC	75	73	6,145	1188	154	26	464	26	154	361
GHA	79	77	23,640	326	60	50	55	40	45	75
KEN	29	29	30,992	94	6	16	23	29	3	16
TAN (I)	33	33	8,128	406	19	97	97	58	39	97
TAN (P)	66	66	18,882	350	7	81	121	40	40	60

Web appendix 6b: Cause-specific antepartum stillbirths rate

SITE	Number of SB	% of SB in antepartum period	Number of antepartum SB	Number of Births	Antepartum stillbirths rates per 1000 births								
					Overall	Congenital malformations	Other specific fetal causes	Maternal medical conditions				Complications of placenta: Antepartum Haemorrhage	Complications of labour and delivery
								Hypertensive disorder of pregnancy	Infections	Other medical conditions	Accidents/Injuries		
BAN	1068	42%	450	27,363	**16.4**	0.0	0.0	7.3	1.2	0.2	0.0	1.5	6.1
IND (H)	798	39%	309	35,798	**8.6**	1.0	1.1	2.4	2.1	0.2	0.3	1.5	0.0
IND (U)	1479	57%	841	39,292	**21.4**	0.7	2.6	2.5	4.9	2.7	0.8	5.9	1.3
PAK (M)	1211	59%	710	28,273	**25.1**	1.1	0.2	18.0	2.3	0.3	0.2	3.2	0.0
PAK (K)	675	63%	424	17,864	**23.7**	1.8	0.9	13.1	2.1	0.1	0.9	4.7	0.1
DRC	155	51%	79	6,300	**12.5**	0.0	0.0	0.9	8.9	0.2	0.0	1.6	0.9
GHA	654	64%	418	24,294	**17.2**	0.3	0.8	2.2	6.9	2.5	0.1	3.9	0.4
KEN	233	46%	108	31,225	**3.5**	0.3	0.0	0.4	1.9	0.1	0.0	0.4	0.3
TAN (I)	123	80%	98	8,251	**11.9**	0.3	0.0	4.3	4.9	0.3	0.0	2.1	0.0
TAN (P)	498	79%	394	19,380	**20.3**	0.3	0.2	9.2	4.0	2.3	0.0	4.2	0.0
ZAM	441	58%	254	25,523	**9.9**	0.4	1.7	1.5	4.6	0.1	0.0	0.3	1.3

Web appendix 6b: Cause-specific intrapartum stillbirths rate

SITE	Number of SB	% of SB in intrapartum period	Number of Intrapartum stillbirths	Number of births	Intrapartum stillbirths rates per 1000 births								
								Maternal medical conditions					
					Overall	Congenital malformations	Other specific fetal causes	Hypertensive disorder of pregnancy	Infections	Other medical conditions	Accidents /Injuries	Complications of placenta: Antepartum Haemorrhage	Complications of labour and delivery
BAN	1068	58%	618	27363	**22.6**	0.1	0.0	5.4	0.5	0.3	0.0	0.9	15.3
IND (H)	798	61%	489	35798	**13.7**	1.7	1.0	1.6	1.5	0.2	0.0	1.9	5.7
IND (U)	1479	43%	638	39292	**16.2**	0.3	3.2	0.9	1.6	1.0	0.1	1.6	7.6
PAK (M)	1211	41%	501	28273	**17.7**	0.5	0.3	5.8	0.9	0.1	0.1	1.8	8.3
PAK (K)	675	37%	251	17864	**14.1**	0.2	1.0	4.0	0.5	0.0	0.3	1.3	6.8
DRC	155	49%	76	6300	**12.1**	0.0	0.2	0.0	1.9	0.0	0.0	1.0	9.0
GHA	654	36%	236	24294	**9.7**	0.1	0.1	0.3	1.1	0.2	0.0	1.1	6.8
KEN	233	54%	125	31225	**4.0**	0.1	0.3	0.3	0.5	0.0	0.0	0.6	2.2
TAN (I)	123	20%	25	8251	**3.0**	0.0	0.0	0.9	0.6	0.0	0.0	0.3	1.2
TAN (P)	498	21%	104	19380	**5.4**	0.1	0.1	0.4	0.2	0.0	0.0	1.1	3.5
ZAM	441	42%	187	25523	**7.3**	0.1	1.0	0.1	0.5	0.0	0.0	0.4	5.1

Web appendix Table 6c: Cause-specific neonatal mortality rate per 1000 livebirths

Number			Neonatal mortality rate per 1000 livebirths								
SITE	Neonatal death	Livebirths	Overall	Preterm Birth	Perinatal Asphyxia	Severe Neonatal Infection	Neonatal Diarrhoea	Neonatal Tetanus	Congenital malformation	Accident /Injuries	Other Perinatal causes
BAN	995	26295	**37.8**	5.9	13.9	17.5	0.0	0.2	0.3	0.0	0.1
IND (H)	1399	35000	**40.0**	10.2	15.7	10.6	0.2	0.1	2.9	0.1	0.2
IND (U)	1575	37813	**41.7**	5.4	16.1	16.1	0.3	0.4	1.6	0.3	1.5
PAK (M)	1269	27062	**46.9**	9.1	20.0	15.1	0.2	0.1	1.7	0.0	0.7
PAK (K)	862	17189	**50.1**	11.4	20.2	14.7	0.5	0.2	2.5	0.2	0.5
DRC	173	6145	**28.2**	7.1	5.8	14.2	0.0	0.3	0.0	0.0	0.8
GHA	687	23640	**29.1**	8.5	10.5	8.1	0.0	0.2	0.8	0.1	0.8
KEN	397	30992	**12.8**	5.0	4.0	3.5	0.1	0.0	0.2	0.0	0.0
TAN (I)	221	8128	**27.2**	3.8	10.6	12.3	0.0	0.0	0.4	0.0	0.2
TAN (P)	303	18882	**16.0**	2.1	7.5	5.0	0.0	0.0	0.6	0.1	0.8
ZAM	363	25082	**14.5**	4.0	3.9	5.4	0.2	0.1	0.5	0.1	0.3

Web figure 1. Meta-analysis forest plots for: (a) maternal mortality ratios; (b) stillbirth rates; and (c) neonatal mortality rates

i. Maternal mortality ratio (per 100 000 live births)

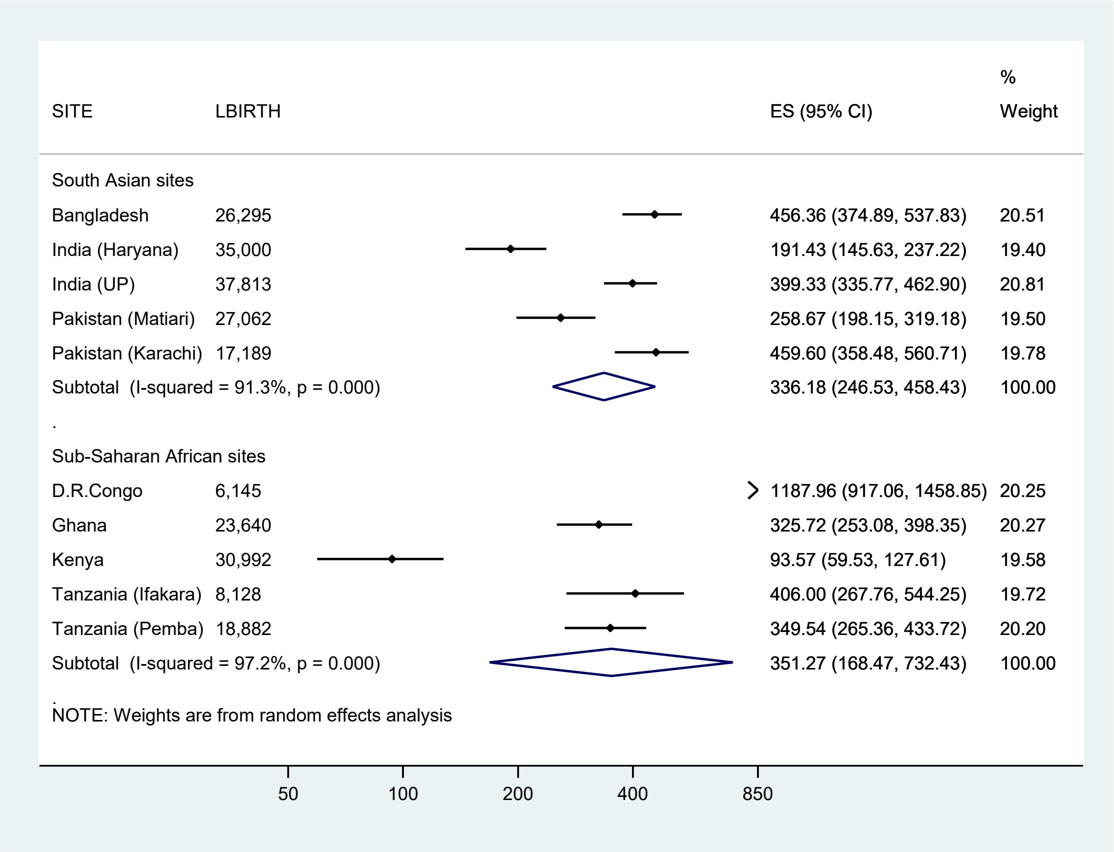

ii. Stillbirth rate (per 1000 births)

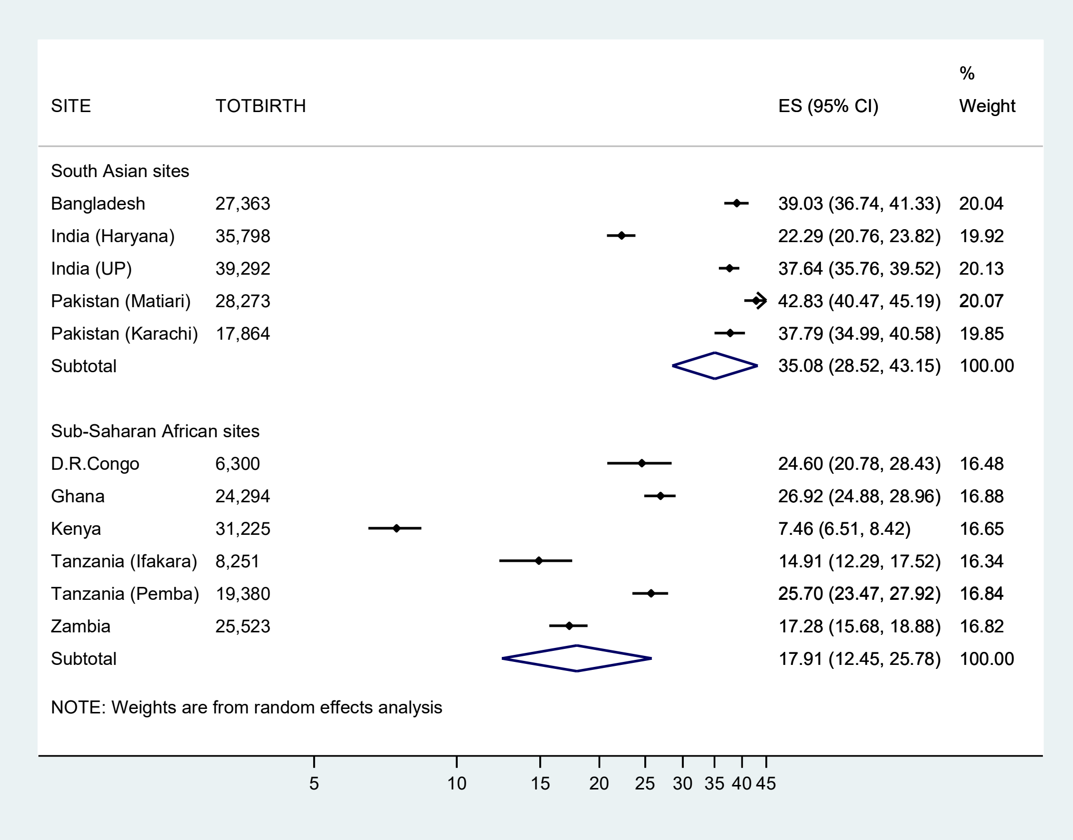

iii. Neonatal mortality rate (per 1000 live births)

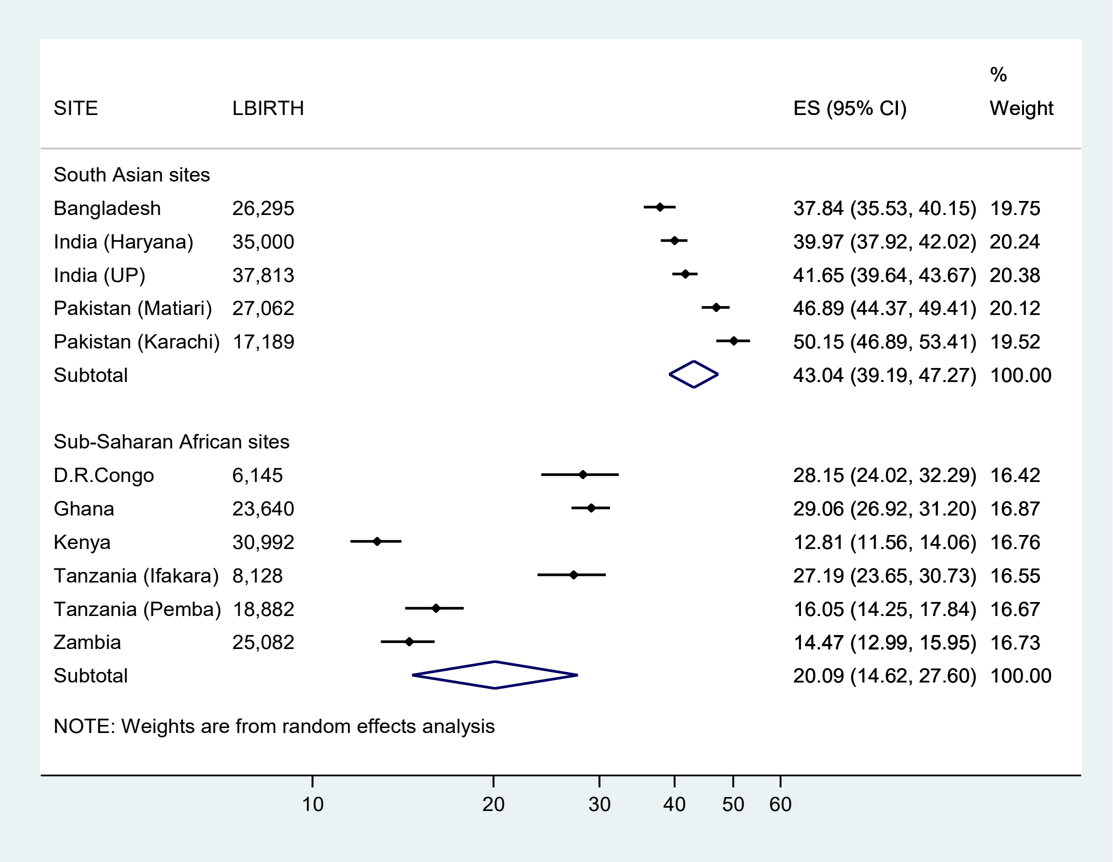

Web figure 2. Timing of deaths in each region

Pregnancy related deaths, stillbirths and neonatal deaths: distribution shown separately by mortality

a) South Asia

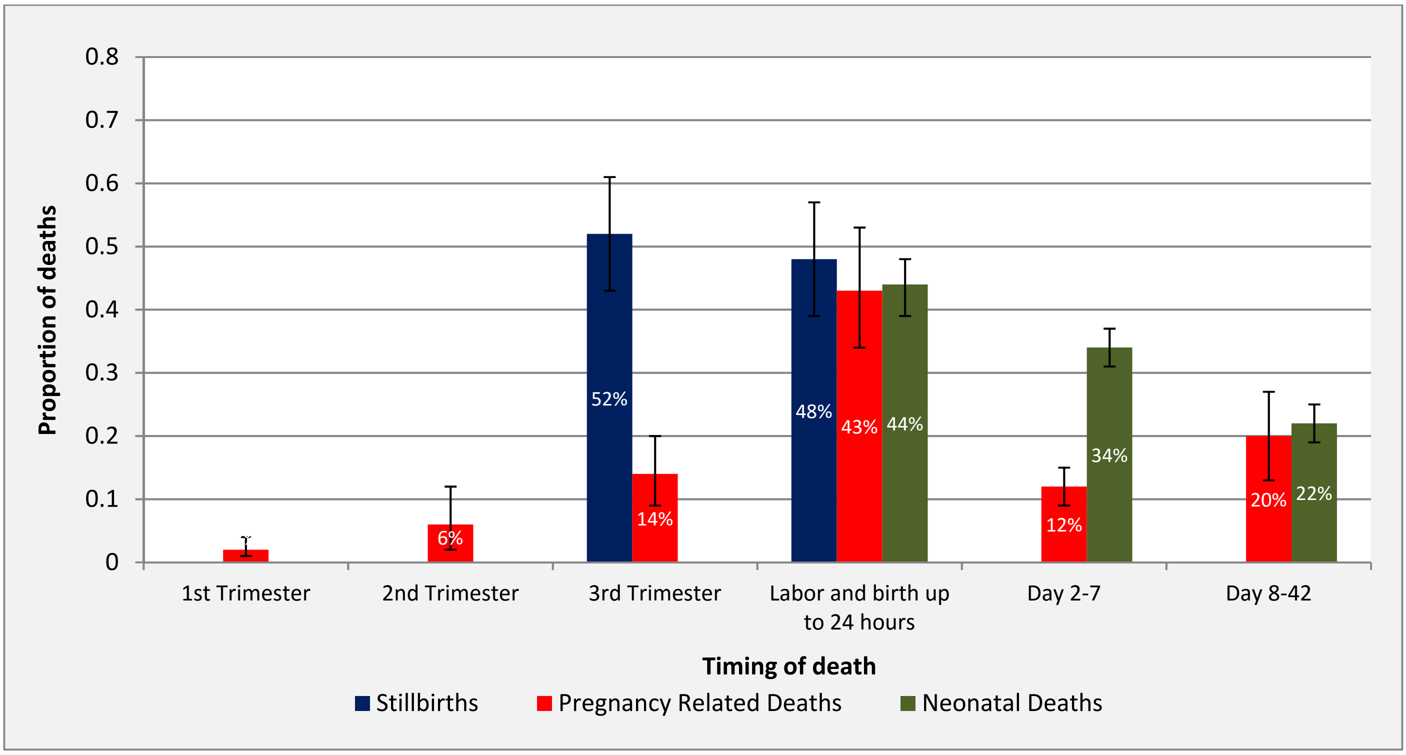

b) sub -Saharan Africa

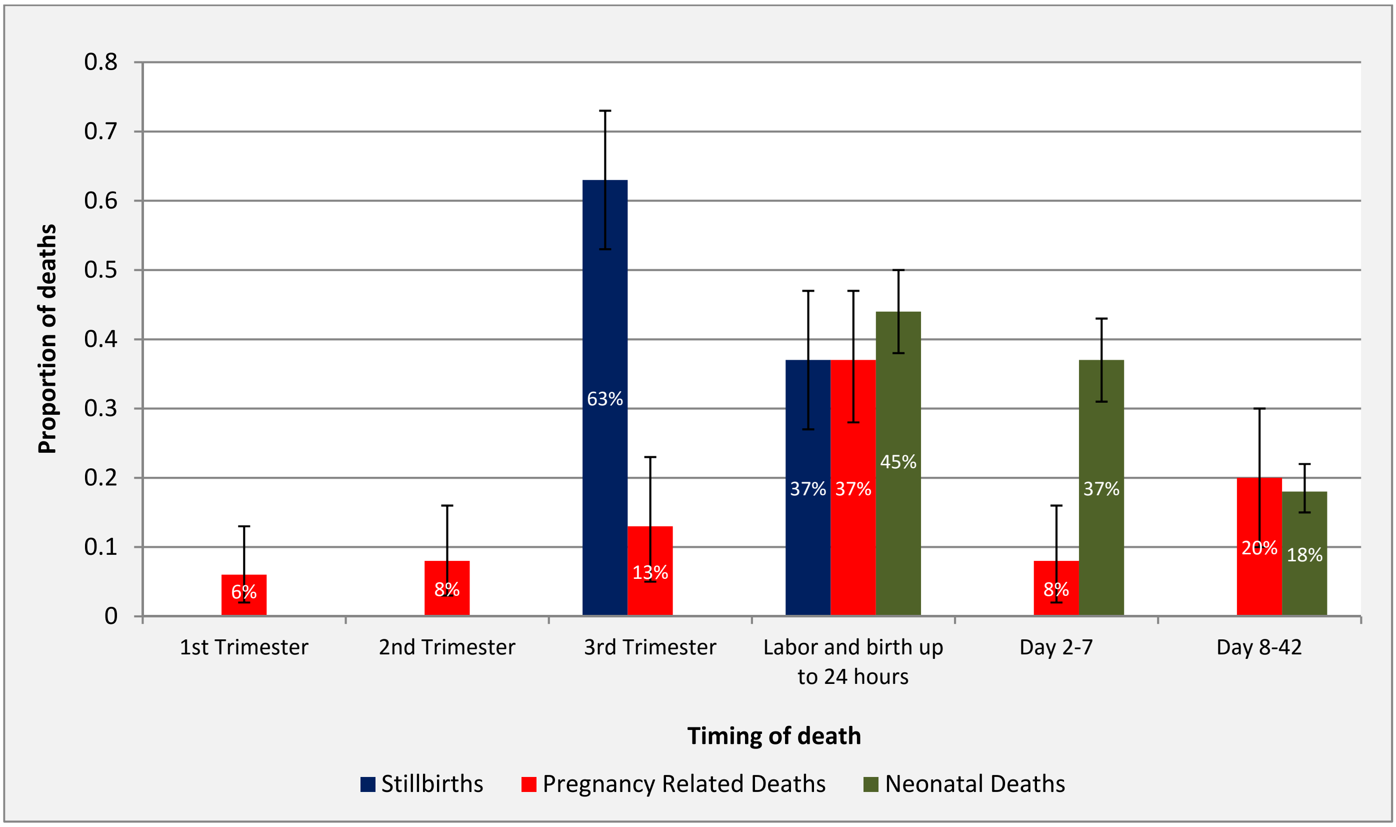

Web figure 3. Relationship of stillbirth rates and neonatal mortality rates with percentage of births in health facilities

(i) Stillbirth rates and percentage of births in health facilities by site

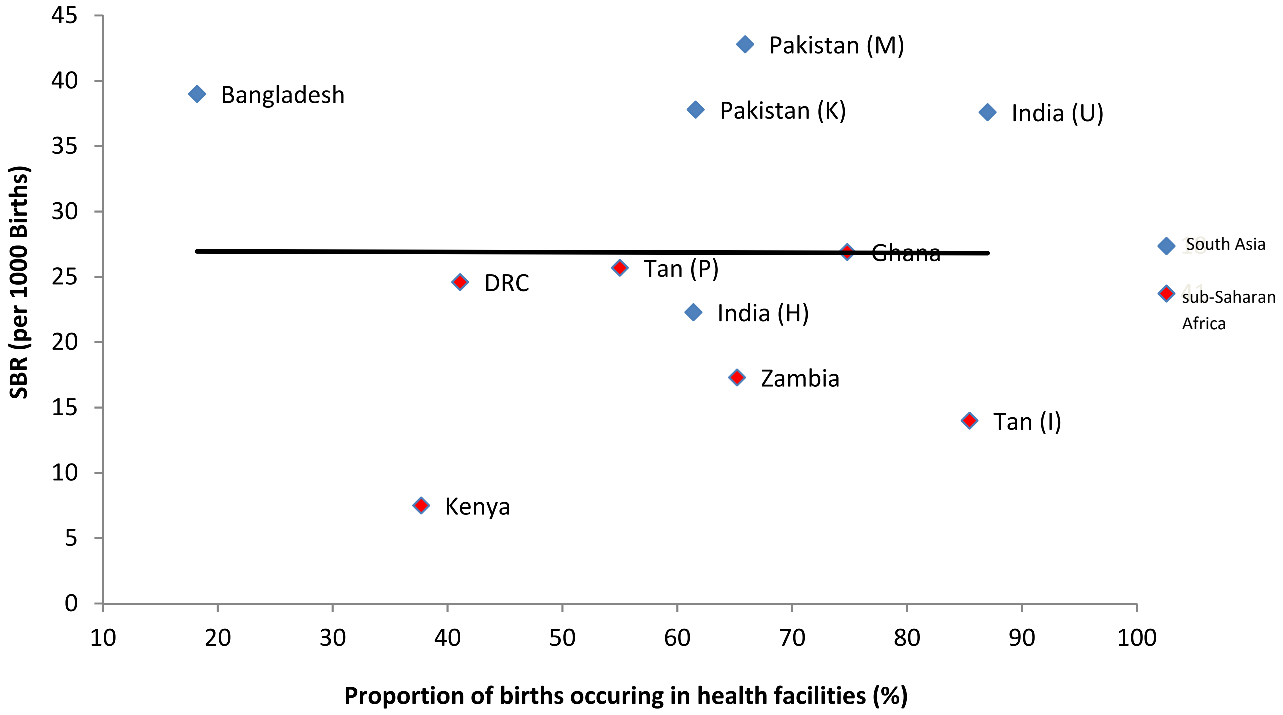

R^2 =0.000, p=0.992

(ii) Neonatal mortality rates and percentage of births in health facilities by site

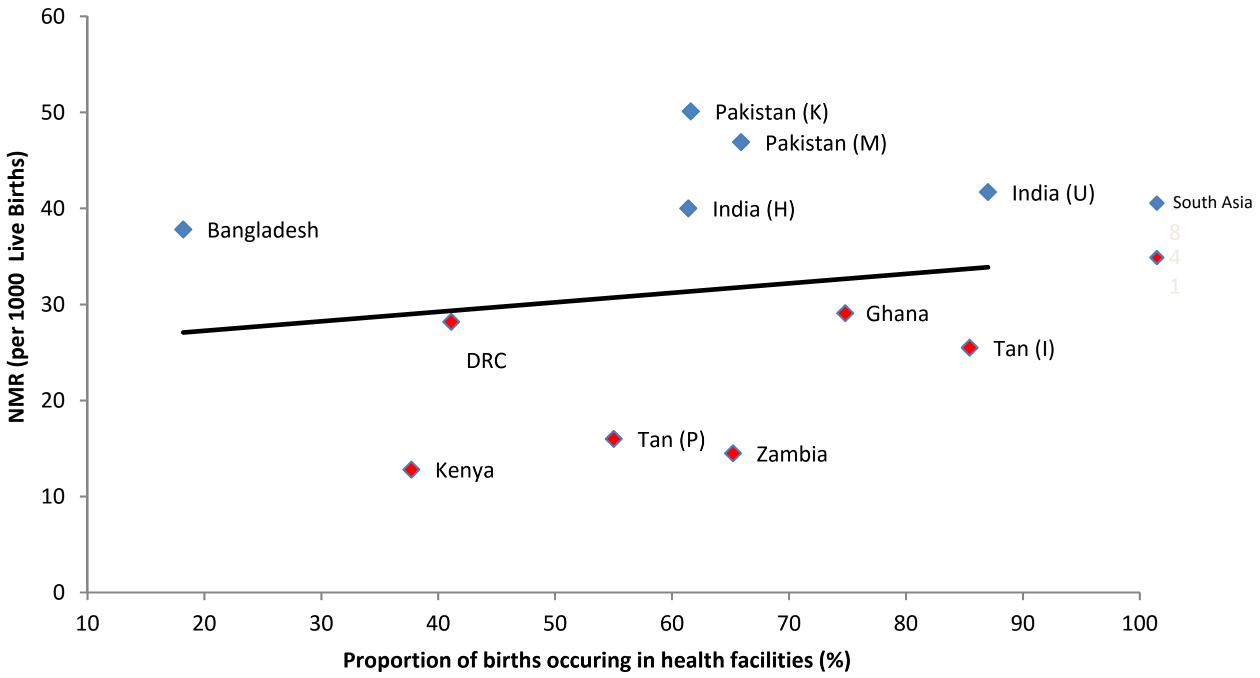

R^2 =0.024, p=0.646

Web figure 4. Relationships of stillbirth rates and neonatal mortality rates with percentage mothers with any schooling

(i) Stillbirths and percentage of mothers with any schooling by site

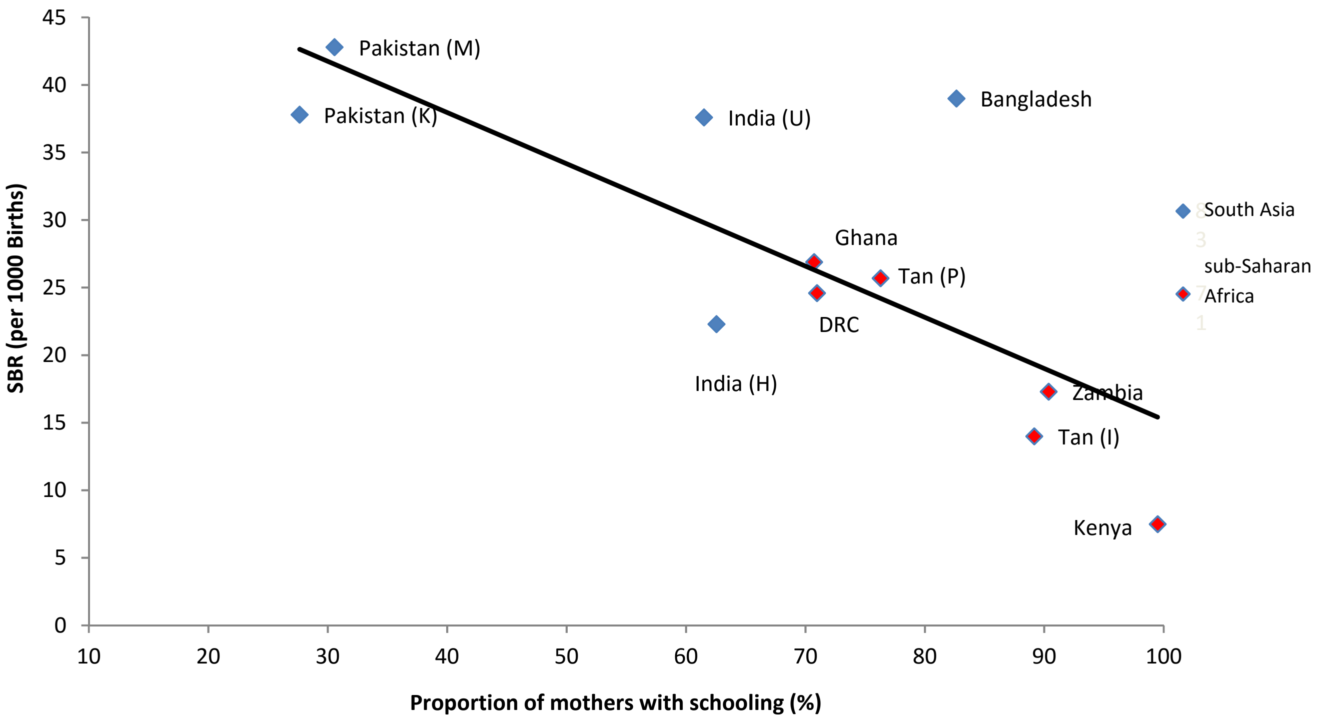

R^2 =0.5901, p= 0.006

(ii) Neonatal mortality rate and percentage of mothers with any schooling by site

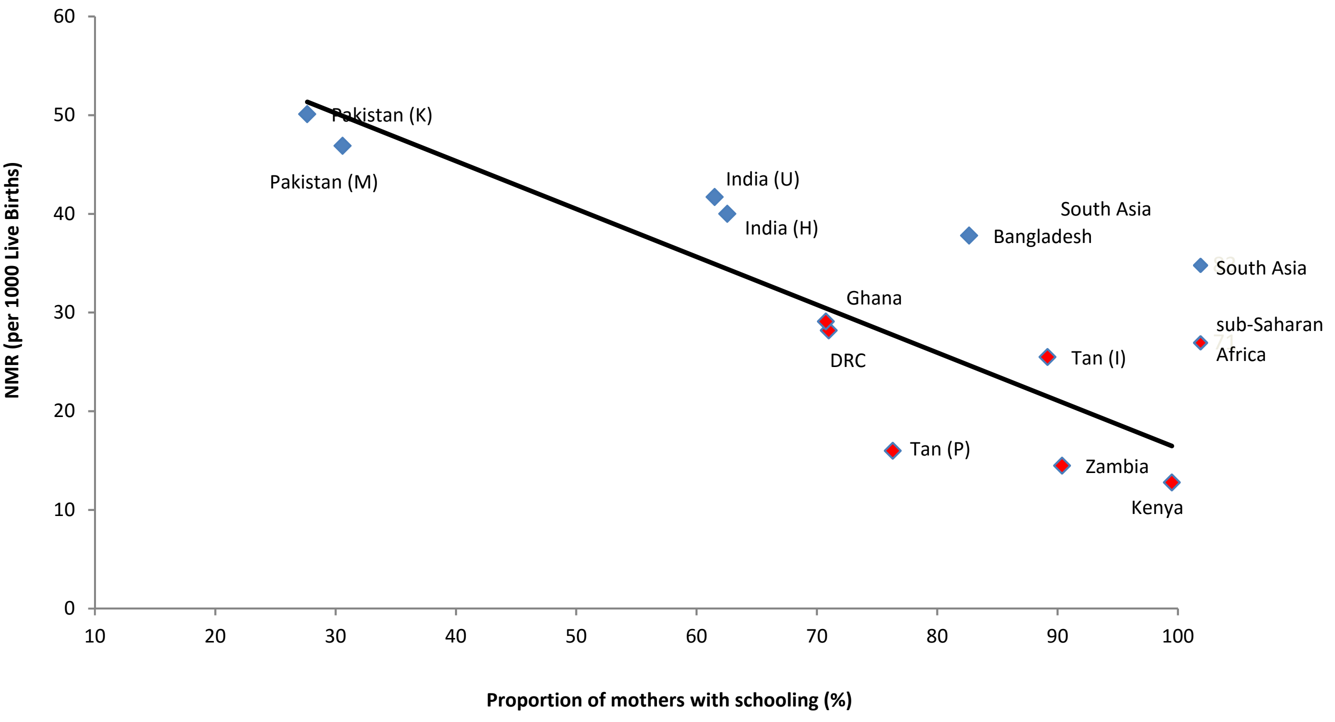

R^2 =0.7249, p=0.001
